# Easy ROMP of Quinine Derivatives Toward Novel Chiral Polymers That Discriminate Mandelic Acid Enantiomers

**DOI:** 10.3390/polym17121661

**Published:** 2025-06-15

**Authors:** Mariusz Majchrzak, Karol Kacprzak, Marta Piętka, Jerzy Garbarek, Katarzyna Taras-Goślińska

**Affiliations:** Faculty of Chemistry, Adam Mickiewicz University in Poznań, 8 Uniwersytetu Poznańskiego Str., 61-614 Poznań, Poland

**Keywords:** ROMP polymerization, quinine, cinchona alkaloids, Grubbs catalysts, enantiomer recognition, mandelic acid, click chemistry, (poly)norbornene

## Abstract

A novel and general approach to the practical ROMP polymerization of cinchona alkaloid derivatives providing novel hybrid materials having quinine attached on a poly(norbornene-5,6-dicarboxyimide) matrix is presented. The concept involves an easy modification of quinine (in general, any cinchona alkaloid) toward clickable 9-azide that reacts with *N*-propargyl-*cis*-5-norbornene-*exo*-2,3-dicarboxylic imide in Cu(I)-catalyzed Huisgen cycloaddition (click chemistry). The resulting monomers undergo a controllable ROMP reaction that leads to novel polymers of a desired length and solubility. This sequence allows for the facile preparation of a regularly decorated polymeric material having one quinine moiety per single mer of the polymer chain inaccessible using typical immobilization methods. A poly(norbornene-5,6-dicarboxyimide) type of polymeric matrix was selected due to the high reactivity of the *exo*-norbornene motif in Ru(II)-catalyzed ROMP and its chemical and thermal stability as well as convenient, scalable access from inexpensive *cis*-5-norbornene-*exo*-2,3-dicarboxylic anhydride (‘one-pot’ Diels–Alder reaction of dicyclopentadiene and maleic anhydride). An appropriate combination of a Grubbs catalyst, Ru(II) (G1, G2), and ROMP conditions allowed for the efficient synthesis of well-defined soluble polymers with mass parameters in the range *Mn* = 2.24 × 10^4^ – 2.26 × 10^4^ g/mol and *Mw* = 2.90 × 10^4^–3.05 × 10^4^ g/mol with good polydispersity, *Đ_M_* = 1.32–1.35, and excellent thermal stability (up to 309°C T_d10_). Spectroscopic studies (NMR and electronic circular dichroism (ECD)) of these products revealed a linear structure with the slight advantage of a *trans*-configuration of an olefinic double bond. The resulting short-chain polymer discriminates mandelic acid enantiomers with a preference for the (*R*)-stereoisomer in spectrofluorimetric assays. This concept seems to be rather general with respect to other molecules dedicated to incorporation into the poly(norbornene-5,6-dicarboxyimide) chain.

## 1. Introduction

**Figure 1:**
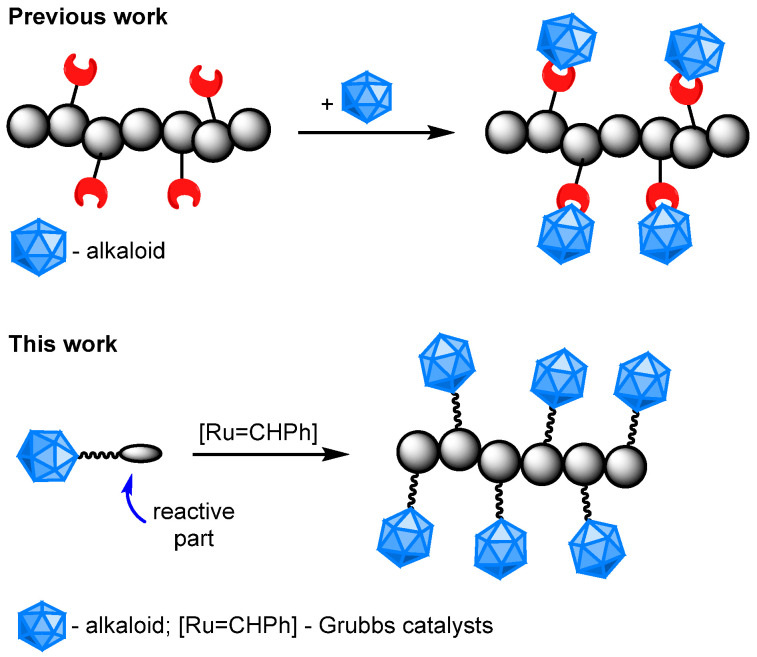
Commonly used immobilization strategies of cinchona alkaloids (CAs) on the existing polymer or support (upper panel) and by the ROMP of a properly designed CA monomer toward directly decorated polymers (bottom panel).

Recently, there has been growing interest in the chemistry and technology of specific chiral polymeric materials dedicated to modern stereoselective synthesis (solid-phase synthesis), the efficient separation of racemic mixtures or the detection of chiral compounds. Cinchona alkaloids (CAs), comprising quinine and quinidine as well as their numerous derivatives, occupy an exceptional position here, being privileged catalysts, ligands and selectors in stereoselective synthesis and chirotechnology [1,2,3,4,5]. They are also frequently used for the construction of diverse chiral molecular systems for enantiodiscrimination and enantioseparation dedicated to liquid chromatography, capillary electrophoresis, membrane separation and other applications [3,4]. Some of their derivatives can be used as chiral ligands for transition metal catalysis [6] and organo-/metal cooperative catalysis [7], but a very important application of cinchona alkaloids is their use as chiral organocatalysts [5,8,9,10,11]. Surprisingly, most CA applications use a homogeneous rather than immobilized format, although the latter offers stability, facile catalyst/selector re-use and compatibility with automated flow chemistry or separation/detection systems. Polymerization techniques of CA must be dedicated to preserving their chemical and stereochemical integrity and secure catalytic or enantiodiscrimination functionality. The proper selection of chemistries allows for the convenient control of CA polymeric architectures, their solubility, thermal stability and the density of the ligand [12]. There are two major strategies for the preparation of cinchona alkaloid-based polymers. In the first, alkaloids are introduced into pendant arms of an existing polymer [13,14,15]. The second strategy uses a dedicated alkaloid-containing monomer for polymerization, resulting in the formation of a polymeric main chain that contains one CA moiety per mer, according to Figure 1.

In this study, we present efficient and convenient methods for the preparation of new, highly reactive quinine–norbornene monomers that undergo facile and fully controlled ROMP toward new chiral, linear and soluble hybrid polymeric materials having enantiomeric recognition ability.

## 2. Materials and Methods

The chemicals were obtained from the following sources: dichloromethane (DCM, 99.8%), 1,2-dichlorobenzene (>99%), maleic anhydride (MA, 99%), acetic anhydride (>99.5%), hexane (99.5%), *N,N*-diisopropylethylamine DIPEA (>99%), dicyclopentadiene (DCPD, >95%), acetonitrile (99.5%), copper iodide (CuI, >99%), CDCl_3_ (>99.8%), sodium azide (NaN_3_, >99%), Grubbs catalyst first-generation [RuCl_2_(=CHPh)(PCy_3_)_2_]—(G1st) and Grubbs catalyst second-generation [RuCl_2_(=CHPh)(PCy_3_)(IMesH_2_)]—(G2nd) were purchased from Sigma-Aldrich (Merck) Poznań, Poland, and Celite^®^ 545 and silica gel 60 from Fluka. Quinine (>98%) and hydroquinine (>95%) were purchased from Buchler GmbH (Quickborn, Germany). Other solvents, e.g., petroleum ether, pentane (99%), hexane (99%), acetone (>98%) and magnesium sulfate (MgSO_4_), were bought from domestic suppliers. The ligands [(8*S*,9*S*)-9-azido-(9-deoxy)dihydroepiquinine] (**3**) and [(8*S*,9*S*)-9-azido-(9-deoxy)-epiquinine] (**4**) were prepared by *Mitsunobu* inversion according to the literature procedure [16]. All syntheses and catalytic tests were carried out under an inert argon atmosphere. Synthetic protocols for anhydride *exo*-**1** and monomers **4** and **5** are provided in detail in ESI.

### 2.1. Synthetic Part

#### 2.1.1. General Procedure for Catalytic Screening of ROMP Polymerization

The ROMP reactions were carried out in argon using a conventional vacuum–argon technique (Schlenk line). Monomer **5** or **6** (9 × 10^−5^ mol, 49.6–49.7 mg) was weighed in a two-neck, small round-bottom flask (5 mL) equipped with a magnetic stirrer, a reflux condenser and ‘*bubbler*’. Then, 1 mL of DCM was added, the mixture was stirred and the flask was placed in an oil bath at 45 °C. After complete dissolution of the substrate, 4.5 × 10^−7^ mol (or 9 × 10^−7^ mol, [mon]:[cat.] = 100:1) of Grubbs 1st- or 2nd-generation catalyst was added in one portion. G1 (or G2) species should be added to the reaction mixture as a solution at the specified concentration. The molar ratio of the components of the reaction mixture was as follows: [cat.]:[mon.] = 1:200, 1:100 or 1:50. After 3–9 h, the reaction was terminated by adding 100 µL ethyl vinyl ether. The crude product was isolated by precipitation three times in cold hexane, collected by suction and dried. NMR and GPC were used for the characteristics of polymers and to optimize the process condition.

#### 2.1.2. General Procedure for Synthesis of Polymers P1 and P2

In a two-neck round-bottom flask (10 mL) equipped with a magnetic stirrer and reflux condenser, 2.71 × 10^−4^ mol of monomer **5** (0.150 g) or **6** (0.149 g) was weighed. The air was removed and the system was filled with argon using vacuum gas manifold (Schlenk line). Then, 3 mL of DCM was added and the flask was placed in an oil bath at 45–50 °C. After complete dissolution of the substrate, 2.30 mg (2.7 × 10^−6^ mol) of Grubbs 2nd catalyst was added. After 3 h of reflux, reaction was terminated by adding 0.2 mL ethyl vinyl ether. The mixture was filtered through thick glass tissue paper (separating catalyst residues), and crude product was isolated and purified by precipitation three times in cold hexane. The final products were filtered off and dried under vacuum. Polymers **P1** and **P2** were isolated as white or white-off solids with 90% for **P2** (133 mg) and 96% for **P1** (143 mg) yields, respectively.

## 3. Results

### 3.1. Synthesis of ROMP-Reactive Exo-Norbornene-Quinine Monomers

**Scheme 1:**
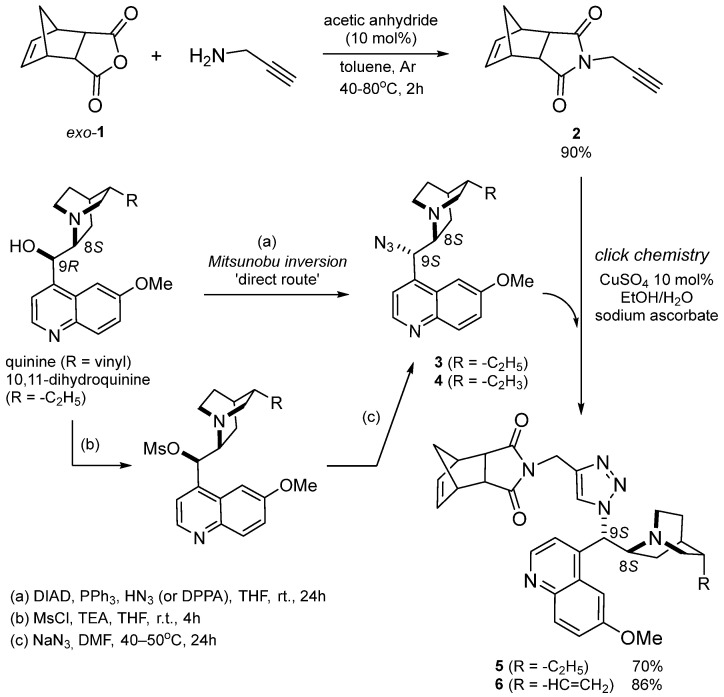
General synthetic way of ROMP-reactive quinine-*exo*-norbornene monomers **5**–**6**.

The norbornene exo-diastereoisomeric derivatives exhibit higher reactivity than the endo-isomers toward ring-opening metathesis polymerization (ROMP) reactions in Ru(II) catalysis because of lower steric hindrance between the growing polymer chains and the incoming monomer [17,18,19,20,21]. As we recently optimized a multigram, reliable and chromatography-free protocol for the preparation of crystalline *cis*-5-norbornene-*exo,exo*-2,3-dicarboxylic anhydride (exo-**1**) with extremely high purity (>99%, after quadruple recrystallization from cold acetone), 1 was prepared via one-pot high-temperature dicyclopentadiene (DCPD) cracking (at 180°C) and subsequent Diels–Alder cycloaddition of formed cyclopentadiene with maleic anhydride (for details, see SI). We wanted to investigate the possibility of exo-anhydride **1** for the practical synthesis of ROMP-reactive monomers containing cinchona alkaloids and then test their potential for the synthesis of respective alkaloid-decorated polymers. The assumed synthetic strategy involved the use of **1** for the preparation of clickable *N*-propargyl-*cis*-5-norbornene-*exo,exo*-2,3-dicarboxylic imide (**2**) via a comparable catalytic protocol, as previously reported [22]. Imide **2,** which is a general monomer intermediate that can be further decorated by any azide-bearing ligand in well-known click chemistry methodology, has been obtained in high isolated yields exceeding 90% and high purity (GCMS 99%), according to Scheme 1.

As an azido-functionalized Cinchona alkaloid, derivatives 9-azido-(9-deoxy)-epidihydroquinine **3** and 9-azido-(9-deoxy)-epiquinine **4** were chosen. These azides are easily accessible through two synthesis routes: two steps, involving synthesis of quinines 9-O-mesylates and their subsequent nucleophilic substitution with sodium azide (NaN_3_) [23,24], as well as the direct Mitsunobu-type inversion reaction described by Brunner [25]. Both methods cleanly replaced the 9-(R)-hydroxy group of quinine or 10,11-dihydroquinine for 9-(S)-configurated azide (S_N_2 inversion) and resulted in isolation yields of 80–84% for **4** and 90–92% for **3,** as reported in earlier work [16]. In the final stage, the synthesis of monomers **5** and **6** was completed using the Cu(I)-catalyzed azide alkyne Huisgen cycloaddition (click chemistry) of azides (**3**, **4**) [26] and alkyne **2**. The Sharpless protocol (CuSO_4_/sodium ascorbate) was used to give respective 1,2,3-triazole derivatives with good yields of 70% for **5** and 86% for **6**, both with high chromatographic purity (>98%), as shown in Scheme 1. Those products having a norbornene–imide matrix and connected via ‘triazole bridge’ quinine-derived alkaloids are soluble in common organic solvents, especially halogen derivatives such as DCM and CHCl_3_.

### 3.2. Polymerization Study

**Scheme 2:**
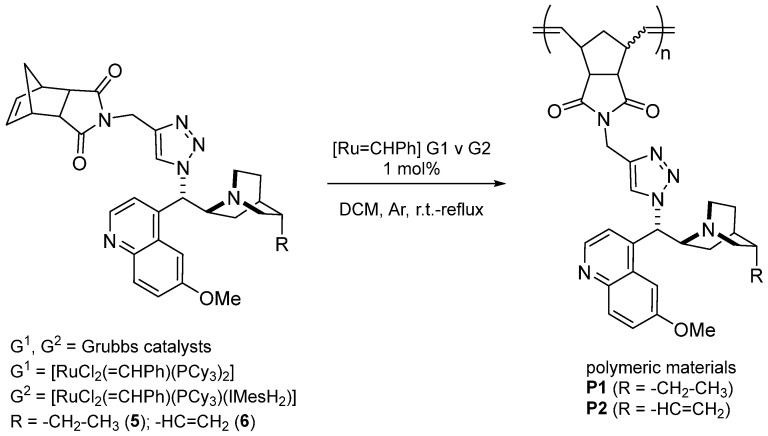
ROMP polymerization of quinine-*exo*-norbornene monomers **5** and **6**.

Compounds **5** and **6** consist of a reactive cyclic norbornene (NB) fragment. This fragment in typical NB derivatives has high ring strain energy of about 27.0–27.2 kcal/mol [27], which makes it particularly susceptible for metathesis ring-opening polymerization (ROMP) reactions catalyzed by Grubbs-type alkylidene ruthenium initiators. To exclude the unwanted self-metathesis of the vinyl group in quinine, initially, the catalytic assays were conducted for parent quinine with G1 and G2 (in molar amounts [cat.]:[mon.] = 1:100, i.e., 1 mol% per mer) in DCM under reaction conditions. The ^1^H NMR spectra were analyzed in detail, and the absence of a diagnostic singlet in the 6.2–7.1 ppm range from the hydrogen of the vinylene fragment (-*H*C=C*H*-) confirmed that no reaction occurred (see Figures S25 and S26 at ESI). Having monomers **5** and **6** in hand, several preliminary catalytic tests were carried out to optimize the reaction conditions, which involved ruthenium(II) catalysts (G1 or G2), catalyst loading and temperature, according to the Scheme 2.

**Figure 2:**
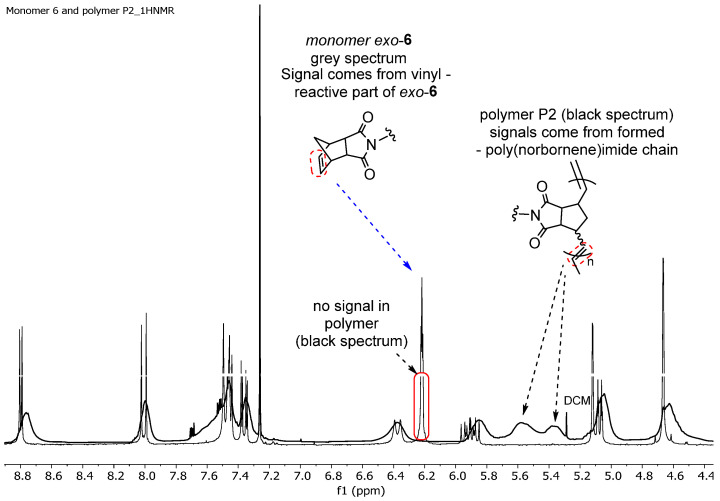
Comparison of two ^1^H NMR spectra of monomer **6** (**---** gray line, QN—denotes quinine) and polymer **P2** (**---** black line) taken in CDCl_3_ at 25 °C.

The progress of ROMP polymerization was monitored using ^1^H NMR. Tests carried out in boiling DCM revealed that the process should be favorably catalyzed with a second-generation Grubbs catalyst (G2, at 0.5 mol%), allowing for the complete consumption of monomers 5 and 6 in 3 h. The use of the G1 catalyst led to a much slower reaction rate, and we could still see monomer residues after 6–7 h. Despite extending the reaction time to 12 h and using a higher amount of G1 catalyst at 2 mol% per monomer (molar ratio = [1]:[50]), traces of the substrate were still detectable. Furthermore, the detailed analysis of the proton spectra confirmed the formation of macromolecular compounds **P1** and **P2** consisting solely of *E*-/*Z*-vinylene fragments in the main chain of the polymer. We detected the complete loss of the signal at 6.22 ppm coming from the vinylene part of monomer **6**(-*H*C=C*H*- norbornene, Figure 2 and see ESI) and the formation of broader signals at 5.36 and 5.58 ppm coming from the inner vinylene part of polymer **P2** (-*H*C=C*H*-cyclopentane part, see ESI). The former is derived from the *Z*-isomer fragment and the latter from the *E*-isomer part of the main chain (percentage composition of isomers: for **P1** *E-*/*Z*- 51/49% and for **P2** *E-*/*Z*- 64/36%).

**Figure 3:**
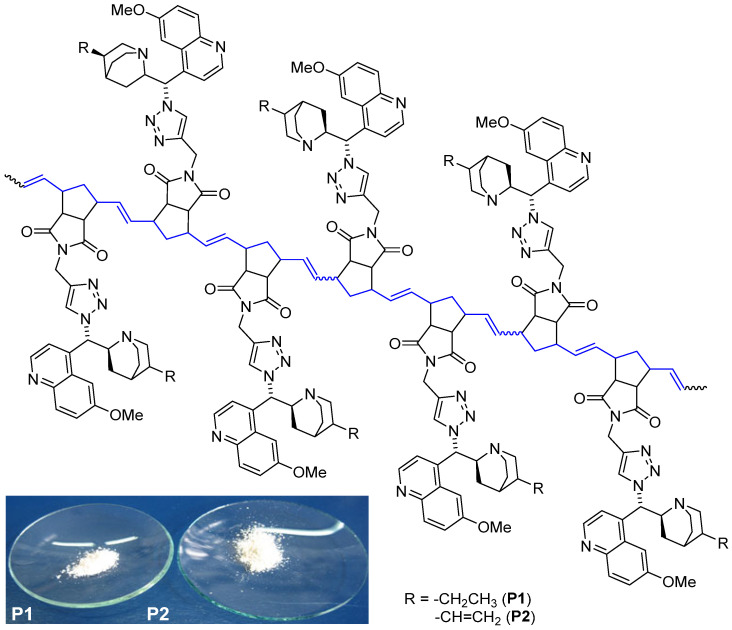
Structure proposal of polymers **P1**/**P2** and pictures of white **P1**, light beige **P2** solid products.

In doing so, we also found that the vinyl group at the 10,11-quinine position in monomer **6** under the mild condition applied was not reactive, as we did not observe any changes in either ^1^H NMR or CD spectra, in line with initial results obtained for quinine itself (see ESI). This suggests no secondary cross-linking of the resulting polymer (**P2**). It is worth mentioning that the vinyl group of quinine is known to be resistant to Ru-catalyzed transformations; its ADMET polymerization required harsh conditions, e.g., high catalyst loading (5% mol) and refluxing over 100 °C for 24 h [28]. Based on this evidence, a reasonable proposal of the polymer structure is presented in Figure 3.

The raw materials **P1** and **P2** underwent purification through successive precipitations (twice) from methylene chloride into cool hexane, and the final fraction was vacuum-dried. These oligomeric materials are white or yellowish solids and exhibit good solubility in CHCl_3_ and CH_2_Cl_2_. It was observed that the use of tetrahydrofuran (THF) for the purification stage of polymers **P1** and **P2** was not recommended due to the material swelling over time. Hence, DCM and CHCl_3_ were used as suitable solvents for the analysis of mass parameters and enantiomer discrimination studies. The basic mass parameters were determined using a standard analysis method via high-pressure gel permeation chromatography (GPC) in DCM showing moderate molecular weight values of soluble polymer **P1** (see in Table 1): *Mn* = 2.24 × 10^4^ g/mol, *Mw* = 2.90 × 10^4^ g/mol and for polymer **P2** *Mn* = 2.26 × 10^4^ g/mol, *Mw* = 3.05 × 10^4^ g/mol. They correspond to mean 40–42 meres in the polymer chain. In addition, the final polymer had good size distributions **P1** *Đ_M_* (*Mw*/*Mn*) = 1.32 (see Figure S18 at ESI) and for **P2** *Đ_M_* (*Mw*/*Mn*) = 1.35 (see Figure S19 at ESI). All data are presented in Table 1.

### 3.3. Characteristics of Polymers—TGA and DSC

The polymers’ thermal stability was determined through thermogravimetric analysis (TGA). All results are presented in Table 2 below. TGA thermal analysis profiles (see in ESI Figures S14–17) showed an initial loss of the minimum weight near 50–55 °C for **P1** (G1) and **P1** (G2) and very similar results for **P2** (G2). In contrast, the mass loss for polymer **P2** (G1) was observed from 95 °C.

All of the studied polymers show unchanged mass with increasing temperature in a range from 25 °C to 160 °C (‘*plato*’ thermal-stable region). The thermal decomposition of polymeric materials occurs in two stages, as shown in the attached TGA curves (refer to ESI) and Table 2. The first stage (1st) takes place between 200 °C and 379 °C, while the second stage (2nd) occurs from 351 °C to 550 °C. It is worth noting that polymer **P2** undergoes a third (3th) decomposition step at temperatures ranging from 504 °C to 770 °C.

The final residue yields were in the range 75–98% at 800 °C. In summary, we found that polymer **P1** was stable up to 242 °C, whereas **P2** was stable up to 219 °C, and a comparison of TGA curves showed no significant differences in the thermal characteristic for the polymers obtained in the presence of Grubbs’ initiators G1 or G2, neither from quinine (vinyl peripherial group) nor dihydroquinine (ethyl group).

The DSC analyses were completed, and the resulting thermal chromatograms exhibited a high degree of similarity for both the **P1** (see Figure S20 at ESI) and **P2** (see Figure S21 at ESI) polymeric materials. No such thermodynamic phenomenon characterized by a change in heat capacity during transition transformations such as crystallization or cross-linking due to uncontrolled thermal polymerization was observed in the thermal diagrams. The existence of such a phenomenon could conceivably serve as evidence for the occurrence of secondary processes. Heating and cooling cycles were conducted in a nitrogen atmosphere, with temperatures ranging from 0 °C to 200 °C.

### 3.4. Characteristics of Polymers—Electronic Circular Dichroism (ECD)

**Figure 4:**
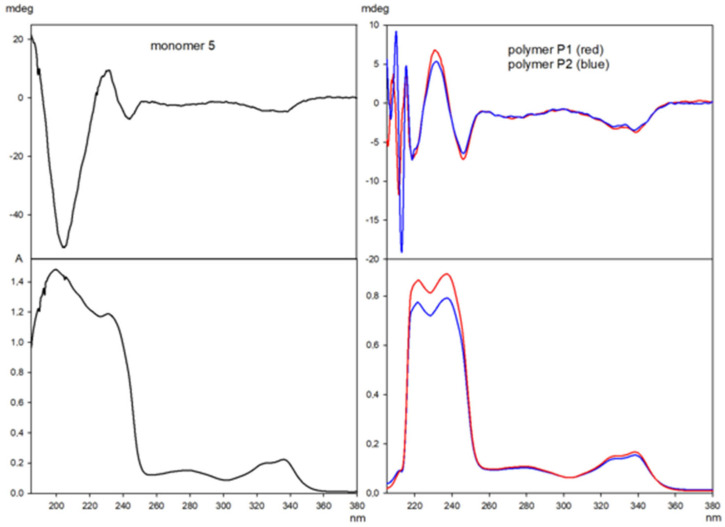
CD and UV spectra of monomer **5** (black line) and polymer **P1** (red line) and **P2** (blue line) in acetonitrile.

Electronic circular dichroism (ECD) spectra were recorded for polymers **P1** and **P2** to investigate the nature of the interactions of polymer chain strands in solution and to exclude the cross-linking of the chain due to the unwanted quinine vinyl group reactivity. ECD is particularly suitable for studying the static or dynamic structure of any chromophores containing a molecule, including polymers [29]. Both the position and the amplitude of the ECD spectrum depend on several factors, where the most important are as follows: the strength of the electronic transition (type of chromophore), the distance between the chromophores, as well as the dihedral angle between the vectors representing the coupled electronic transition dipoles, which correlates with the three-dimensional structure of the studied system [29]. Chiral monomer **5** and polymers **P1** and **P2** synthesized thereof contain three different chromophores active in ECD spectroscopy. 6′-methoxyquinoline is present in the Cinchona alkaloid unit, with *exo*-norbornene imide constituting a polymer chain and 1,2,3-triazole serving as a linker. Circular dichroism spectra of the parent Cinchona alkaloids and their derivatives were studied in detail [30,31]. 6′-Methoxyquinoline is a relatively good chromophore that dominates in their UV and CD spectra. This chromophore in quinine or quinidine usually shows three distinct bands at 313 nm (e: 2500), 268–275 nm (e: 3500) and 226 nm (e: 35,500) in cyclohexane. They were historically designated after Clar as *α, p* and *β* bands and correspond to the ^1^L_b_, ^1^L_a_ and ^1^B_b_ bands in the naphthalene chromophore [30]. The major π-π* transition with a dipole corresponds to the longitudinal direction of the quinoline heterocycle. In this study, both monomer **5** and corresponding polymers **P1**/(G1) and **P1**/(G2) contain the 9-epi-10,11-dihydroquininyl moiety. We assumed that the CD absorption of pendant 6′-methoxyquinoline chromophore could conveniently be used to assess whether there was any way to additionally order the three-dimensional structure in polymers. In particular, the formation of chiral helical domains often observed in other quinoline-based oligomers [32] or polymers [33] was considered worthy of confirming with the aid of CD spectroscopy that is either conclusive or simple. Similarly, the intramolecular reaction of the vinyl group of monomer **6** that potentially leads to a cross-linked polymer, differing in the spatial arrangement of quinoline chromophores, should also be distinct from CD spectra. Two other chromophores present in monomers **5–6** and polymers **P1**, **P2**, namely *exo*-norbornene imide (bicyclo[2.2.1]heptane-2,3-dicarboxylic imide) and 1,2,3-triazole, seem to be less efficient in terms of the adsorption coefficient and amplitude of respective Cotton effects. *exo*-Norbornene imide exhibits the two lowest-energy electronic transitions of n-π*-type low intensity located at ca. 250 and 220 nm. This rigid tricyclic system also becomes inherently chiral (CD active) due to the non-planarity of the flexible imide ring [34]. Thus, the CD spectrum of the *exo*-norbornene diimide is a sum of the contribution of chiral substituents installed close to such chromophores and, to a lesser extent, the effect of the non-planarity of imide chromophores. In the case of our molecular systems, the *exo*-norbornene imide is incorporated into a polymer chain being chiral after ROMP reaction and is remotely joined to a chiral Cinchona alkaloid moiety by five consecutive bonds via a 1,2,3-triazole linker (third chromophore, vide infra). It is expected that weak absorption of the *exo*-norbornene imide chromophore will not contribute significantly to the full CD spectra, and its short-wavelength region will be partially obscured by intense quinoline absorption. Similarly, 1,2,3-triazoles having aliphatic substituents (at least one chiral) are weak chromophores with absorption maximum ca. 220 nm and a low absorption coefficient (ε~3900). They give a very small Cotton effect [35]; thus, we assumed that 1,2,3-triazole would also not contribute significantly to the CD spectrum of monomer **5–6** and related polymers **P1** and **P2**. CD and UV spectra of exemplary monomer 5 and two large-molecular-weight polymers **P1** (G1) and **P2** (G2) obtained with the aid of Grubbs first- and second-generation catalysts, respectively, are presented in Figure 4. It is evident from the CD spectra of monomer **5** and polymers **P1** and **P2** that there is a high degree of similarity in the short- and long-wavelength regions. This observation is further corroborated by the registration of the CD spectra of all other polymers from the screening phase. The spectra show at approximately 205 nm as a broad negative band, with additional peaks observed at 232 (+), 244 (−), 277 (−) and 335 (−) nm. These spectra exhibit a high degree of similarity, with no significant disparities (). For monomer **5**, the strongest CD bands are of much lower intensity (for detailed data, see ESI). Polymers **P1** and **P2** exhibited a more complex but concise picture in the low-wavelength region with CD bands located at ca. 212 (−), 216 (+), 219 (−), which can be attributed to the transition increments originating from the rigidity of the polymer (hindered rotation of quinine chromophore within poly(norbornene) chain or, in part, *exo*-norbornene imide). Other features occurring at 231 (+), 246(−), 277 (−) and 338 (−) nm are identical to those in monomer **5**. Similarly, the UV spectra of monomer **5** and polymers **P1** and **P2** are nearly identical, with major bands located at 221 nm, 238 nm, 280 nm, 327 nm and 338 nm. These bands corresponded closely to the Cotton effects observed in the CD spectra. The observed spectral CD and UV features can be most likely assigned to a π-π* transition with a dipole that corresponds to the longitudinal direction of the quinoline chromophore [30].

The identity of CD spectra of monomer **5** and polymers **P1** and **P2** in acetonitrile and the lack of new Cotton effects in polymers lead to the conclusion that the vinyl group in monomer **5** is inactive in ROMP polymerization. Cross-linking will change the spatial orientation of quinoline chromophores in polymer **2,** which should rather be traceable in CD. In addition, the spectra confirm that polymers adopt rather linear than helical topology. This statement agrees with the observed predominant trans-selectivity of ROMP, as determined by NMR. The polymer chain prefers a linear rather than a curved shape, as shown in Figure 3.

### 3.5. Chiral Discrimination of Mandelic Acid Enantiomers by Polymer P2

**Figure 5:**
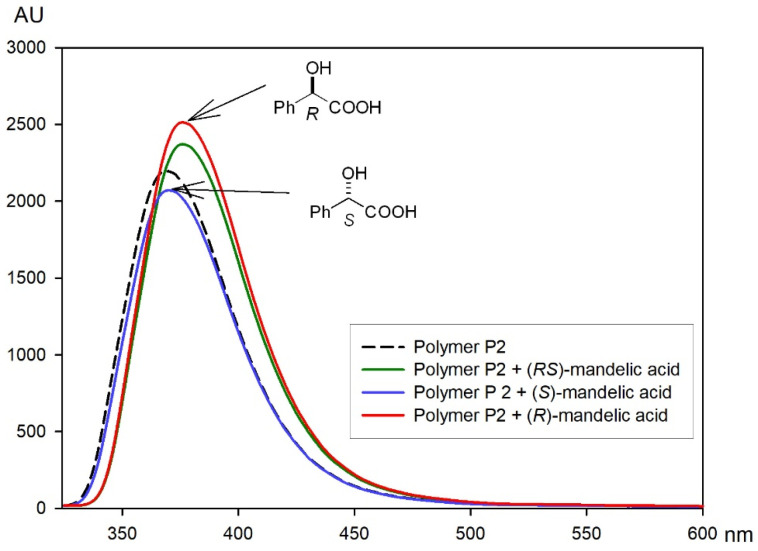
Fluorescence emission of polymer **P2** with *R, S* and racemic mandelic acids allowing their enantio-discrimination (conc. 2.4 × 10^−4^ mM selector, 40 eq. of selected (guest) assuming that 25 mer can theoretically bind 25 molecules of acid, CHCl_3_/MeOH).

Cinchona alkaloids have extraordinary chiral discriminatory power that has been demonstrated in a number of classical diastereoisomeric crystallizations of racemic mixtures, in stereoselective synthesis or enantioseparation [1,2,3,4,13,14,15]. However, in most applications reported so far, monomeric and dimeric Cinchona alkaloid derivatives or their analogues immobilized on solid support were used. Having in hand easy access to polymers **P1** and **P2,** which contain a quinine molecule in each mer, we were interested in testing their enantiodiscrimination properties. Mandelic acid was chosen for this study as a model guest because both native cinchona alkaloids, including quinine [36,37,38] and its derivatives, for example, those with a blocked 9-OH group, e.g., ethers [39] or carbamates [40], were successfully used for the separation of racemic mandelic acids or their enantioselective sensing. The mechanism of enantiodiscrimination in such a pair involving quinine or quinine derivative (selector) and (*R*)- or (*S*)-madelic acid (selected) depends on a number of factors, in particular on the complementarity of the chiral binding site of the selector, the strengths of the relevant non-bonding interactions and the conformational adaptability of the selector molecule. In reported papers, including either the resolution of racemic mandelic acid by crystallization [36,37,38] or enantioselective separation in chiral LC [40], distinct simultaneous multiple contacts involving ion pairing (basic nitrogen atom of quinuclidine part and carboxylic group of acid), hydrogen bonding, and *π*-stacking were suggested for the tightly bounded *R* enantiomer of mandelic acid. For example, Báthori showed that the resolution of mandelic acid by quinine was controlled by ion pairing and intermolecular hydrogen bonds that resulted in conformational changes in protonated quinine and mandelate carboxylate anion [38]. Due to problematic readout of the interaction of **P2** and selected molecules in ^1^H NMR spectra (**P2** has a fairly rich proton spectrum (see ESI) mostly overlapping the selected signals), we decided to use fluorescence spectroscopy for tracing the enantiodiscrimination instead. Fluorescence is a very sensitive technique frequently used in chiral sensing [41], and we assumed that the presence of a quinoline chromophore in quinine (that shows strong emission in both neutral and protonated form) makes it well suited for this task. Thus, polymer **P2** was studied at a 2.4 × 10^−4^ mM concentration in spectral chloroform for fluorescence testing and after the optimization of measurement conditions (see Methods), we found that excitation at 320 nm gave considerably intense emission at 369 nm, with a quantum yield of fluorescence of 0.2. The addition of *R*-mandelic acid added in small aliquots in methanol in a range from 1 to 40 equivalents (note: **P2** consisted of 25–26 quinine mers and each theoretically could bind one acid molecule) led to a higher ratio of *R*-enantiomer and an increase in the emission of fluorescence, as compared with polymer **P2** starting (Figure 5). Moreover, a ca 10 nm batochromic shift in emissions was also observed, suggesting the conformational rearrangement of the selector upon binding the *R*-enantiomer. In contrast to this, (*S*)-mandelic acid used in an identical concentration range did not enhance the intensity or change the position of the emission band in comparison to free polymer **P2**. A competitive experiment was performed with racemic (*R,S*)-mandelic acid, too. At a higher ratio (in total, 20–40 equivalents) of acid, very similar spectra to those observed by *R*-mandelic acid were obtained (Figure 5). This led to the conclusion that **P2** had higher affinity to the *R*-enantiomer, even if *S*- was present in a similar quantity.

Similar tests performed on a simple monomer **5** (at concentration 0.018 M) having an identical molecular architecture comprising quinine linked by 1,2,3-triazole to the norborneneimide with *R*, *S* and *R,S*-racemic mandelic acids, respectively, showed no changes in emission spectra and the band position (see Figure S23 in ESI). This allows us to exclude a non-cooperative chiral discrimination phenomenon and provides some evidence that this rather rigid polymer chain flanked with 1,2,3-triazole linked quinines forms a set of new distinct multi-binding site architecture complementary to the *R*-enantiomer. Thus, the results clearly demonstrate that the new polymer **P2** is capable of the enantiomeric recognition of mandelic acid in CHCl_3_ solution, with a significant preference for *R*-enantiomer [36,37,38,39,40].

### 3.6. Conclusions

In summary, a novel strategy for the practical synthesis of the Cinchona alkaloid-based polymeric materials with enormous dense packing of the quinine ligand is demonstrated, using the advantages of the high reactivity of *exo*-norbornene monomers in ROMP. To the best of our knowledge, this is the first example of ROMP polymerization in the field of Cinchona alkaloid-related materials. This novel route gives soluble materials **P1–P2** with very good isolation yields (70–93%) and high purity. Moreover, these polymeric products **P1** and **P2** have linear, regular structures with alternating arranged quinine-linked units and moderate molecular weight (*Mn*) in a 2.23 × 10^4^–2.26 × 10^4^ (g/mol) range, ensuring solubility and a quite narrow *Đ_M_* index (1.32–1.35). Polymer **2** recognizes enantiomers of mandelic acid in solution by using fluorescence emission.

These readily amenable, accessible and highly dense quinine-packed materials are not easily accessible using classical immobilization methods and are currently studied in our group toward analytical enantiomer discrimination and separation.

## Figures and Tables

**Table 1:** GPC mass parameters of polymeric materials **P1** and **P2**.

Polymer (Catalyst Used)	*Mn* ^a^ (g/mol) ×10^4^	*Mw* ^a^ (g/mol) ×10^4^	*Đ_M_* (*Mw*/*Mn*)	Yield (%) ^b^
**P1** (G2)	2.2365 ^c^	2.9020 ^c^	1.32	96
**P2** (G2)	2.2618 ^c^	3.0494 ^c^	1.35	89

^a^ GPC conditions: dichloromethane (DCM), flow rate 0.80 mL/min, temp. 25–40 °C; the volume of the injection 100 μL; ^b^ after isolation; ^c^ GPC analysis results multiplied by the factor ×0.5, recalculated according to literature recommendations are the actual mass parameters (see ESI).

**Table 2:** Thermogravimetric analysis data of polymeric materials **P1**/**P2**.

Polymer (Catalyst Used)	T_d_^5^ (°C) ^a^	T_d_^10^ (°C) ^a^	1st Step of Thermal Decomp. (Weight Resid. (%)) Top Temp. ^a^ (°C)	2nd Step of Thermal Decomp. (Weight Resid. (%)) Top Temp. ^a^ (°C)	3th Step of Thermal Decomp. (Weight Resid. (%)) Top Temp. ^a^ (°C)	Total Weight Residue (%)
**P1** (G1) (Figure S14 ESI)	242	285	200–390 (38) Max. 306	390–550 (22) Max. 431	----	75
**P1** (G2) (Figure S15 ESI)	286	299	257–367 (22) Max. 301.0	351–509 (21) Max. 447	504–769 (48) Max. 594	92
**P2** (G1) (Figure S16 ESI)	219	287	231.7–379.2 (45) Max. 315.5	375–531 (30) Max. 423	----	80
**P2** (G2) (Figure S17 ESI)	297	310	271–379 (22) Max. 312	382–542 (33) Max. 450	----	76

^a^ Thermal conditions of the TGA method used: N_2_, 10 °C/min; 25–900 °C; All temperature degrees and mass loss percentages have been rounded to whole values.

## Data Availability

All experimental data are included in ESI and are available to reviewers upon request.

## Supplementary Materials

The following supporting information can be downloaded at: https://www.mdpi.com/article/10.3390/polym17121661/s1; it contains spectroscopic characterizations, NMR spectra (Figures S1–S13, S25 and S26), thermogravimetric curves (Figures S14–S17), GPC graphs (Figures S19), DCS curves and other analytics (Figures S20 and S21).
