# Supplementary material for: Easy ROMP of Quinine Derivatives Toward Novel Chiral Polymers That Discriminate Mandelic Acid Enantiomers
Source: Polymers (Basel). 2025 Jun 15;17(12):1661. doi: 10.3390/polym17121661 (PMC12197185; doi:10.3390/polym17121661)
Supplement: Supplementary file 1 [file polymers-17-01661-s001.zip › polymers-3687417-supplementary.pdf]

Electronical Supporting Information (ESI)

Easy ROMP of Quinine Derivatives Toward Novel Chiral Polymers That Discriminate Mandelic Acid Enantiomers

Mariusz Majchrzak*, Karol Kacprzak, Marta Piętka, Jerzy Garbarek
and Katarzyna Taras-Goślińska

Faculty of Chemistry, Adam Mickiewicz University in Poznań, 8 Uniwersytetu Poznańskiego st., 61-614 Poznań, Poland
Correspondence: mariusz.majchrzak@amu.edu.pl

Table of contents

I. Experimental part 1
II. Instruments and measurement 1
III. Synthetic part: synthesis procedures and analytic data 3
IV. General procedure for self-metathesis of quinine 6
V. Polymers P1 and P1 analytic data 7
VI. NMR Spectra 9
VII. TGA analysis 16
VIII. GPC analysis and data 18
IX. DSC Spectra and data 20
X. CD analysis table 22
XI. FL emission spectra and data 23
XII. NMR spectra for general procedure for self-metathesis of quinine 25
XIII. References 26

Experimental part

Instruments and measurement

Nuclear magnetic resonance (NMR) spectroscopy: ^{1}H NMR (300, 400 MHz) and ^{13}C NMR (75, 101 MHz) spectra were recorded on a Varian XL 300 MHz spectrometer, Varian VNMR-S 400 MHz spectrometer and Varian 600 MHz spectrometer in $CDCl_3$ solutions. Chemical shifts are reported in (ppm) with the reference to the residue solvents (^{1}H δ_H = 7.26 ppm, ^{13}C δ_C = 77.36 ppm for $CDCl_3$) peak for ^{1}H, ^{13}C. Analytical gas chromatographic (GC) analyses were performed on a Varian Star 3400CX with a DB-5 fused silica capillary column (30 m x 0.15 mm) and TCD. Mass spectra of the substrates and products were obtained by GCMS analysis (Varian Saturn 2100T, equipped with a CP-SLI 6CB capillary column (30 m x 0.25 mm) and an ion trap detector.

Melting points were determined by using *Boetius* instrument. All measurements were taken twice for each sample of monomers **5** and **6**.

High-resolution mass spectroscopic (HRMS) analyses were performed on an AMD-402 mass spectrometer.

An elemental analysis was performed on a Vario Elementary Analyser Vario EL III Instrument (German) three times and then averaged to obtain the final results. The temperature range of analysis was 25–1100°C. The method of analysis involves catalytic combustion of the sample at 1200°C with

gas separation on adsorption columns. Detection of material components was identified by thermal conductivity difference. The duration of a single analysis varied from 10 to 20 minutes with simultaneous determination of the elements: C, H, N, S.

Thin-layer chromatography (TLC) was made on plates pre-coated with plastic sheet with 250 µm thick silica gel (Polygram SilG/UV254, ROTH), and column chromatography was conducted with silica gel 60 (70-230 mesh, Fluka).

Gel permeation chromatography (GPC) analyses were performed using an Agilent HPLC system equipped with RI detector (analysis conditions: mobile phase – dichloromethane (DCM); flow rate 0.80 mL/min; temperature 25-40°C; the volume of the injection 100 µL). Two 2X Plgel 5um mixed C were used for separation. The calibration of the OPTILAB T-reX (Wyatt) was carried out by p.a. grade toluene and normalization with a polystyrene standard of 30,000 g/mol molar mass. The numerical average molecular weight (*Mn*), average molecular weight (*Mw*) and polydispersity index ($Đ_M$ = PDI) were determined by polystyrene standards calibration curve (correlation coefficient: $r^2 = 0.9993563$). In determination of the molecular weight the Astra software should know the amount of the sample introduced for measurements to calculate *dn/dc* of polymer. We measured the *dn/dc* on line with a sample passing through the columns assuming that the polymer did not interact with the columns giving eventually good amount, equal to the value introduced to the software. ASTRA 6.2 software (Wyatt Technology Corporation) was used for data collecting and processing.

The methodology for calculating mass parameters for the GPC method in dichloromethane is as follows: as polynorbornene standards are not commercially available, both mass parameters (*Mn* and *Mw*) of the target polymer should be reported in relation to poly(styrene) standards, with the use of an appropriate correction factor. With regard to this specific type of polymer, different values of correction factor can be found in the literature, depending on the eluent used in GPC measurement. These values range from 0.5 (CH_2Cl_2) to 2.2 (toluene) [1-4]. Furthermore, Bielawski et. all suggest that when THF is used as the eluent, the correction factor is unnecessary.

Thermogravimetric analyses (TGA) were carried out under inert gas (nitrogen, 10mL/min) flow at heating rate of 10°C min^{-1} on a Setaram Setsys 12 instrument with a model Setsoft version 154D data analysis software program (the temperature range of analysis was 25 – 900°C).

Differential Scanning Calorimetry (DSC) Mettler Toledo (Module: DSC1/700/1450 GC10) analyses were carried out under inert gas flow (nitrogen, 35mL/min) at heating/cooling rate of 10°C min-1 from 0 to 200°C. Samples (2-3mg) were placed in 40µL aluminium pans with a pierced lid.

Purification and drying of solvents and deoxygenation of solvents and reagents used in the work were carried out on the basis of standard methods described in the literature. The solvents were stored in Schlenk vessels under argon atmosphere and kept in the previously calcined molecular sieves A4.

Methodology of work in an inert gas atmosphere: reactions in an inert gas atmosphere were carried out using a vacuum gas manifold (Schlenk line). All solvents and liquid reagents used in the reactions were deoxygenated and dried according to standard procedures, described above.

CD and UV-VIS spectra were measured on a Jasco J-810 spectropolarimeter, using quartz cuvette with 1 mm optical path-length in spectral grade acetonitrile (3 accumulations each sample). Each sample ~1.6 mg was dissolved 10 mL of solvent and such solutions were taken to the measurements.

Photophysical measurements: UV-vis absorption spectra were recorded using a Cary 100 spectrophotometer scanning from 600 to 200 nm with 1 nm increments in 1 cm × 1 cm quartz cells. Emission spectra were taken in 1 cm × 1 cm quartz cells on a JASCO FP – 6200 spectrofluorometer (scan speed 240 nm min^{-1}) for the solutions with an absorbance at the excitation wavelength lower than 0.1. Fluorescence quantum yields were determined using quinine sulphate in 0.1 N sulfuric acid as a

standard (Φ = 0.55). Enantiomeric recognition tests by fluorescence were carried out by addition of small aliquots of methanolic solution of appropriate mandelic acid to the solution of **P2** dissolved in $CHCl_3$ at concentration 2,4x10^{-4} mM (not exceeding of 3% of total volume). The UV-VIS and fluorescence emission were measured directly after selectand addition.

Synthetic Part

***Synthesis of cis-5-Norbornene-exo-2,3-dicarboxylic anhydride (exo-AN)* (1)**

A mixture of *endo-/exo*-isomers of norbornene dicarboxylic anhydride was prepared in accordance with the methodology outlined in the literature [5] with a slight modification. Procedure synthesis of title compound **1** was made according to following Scheme S1:

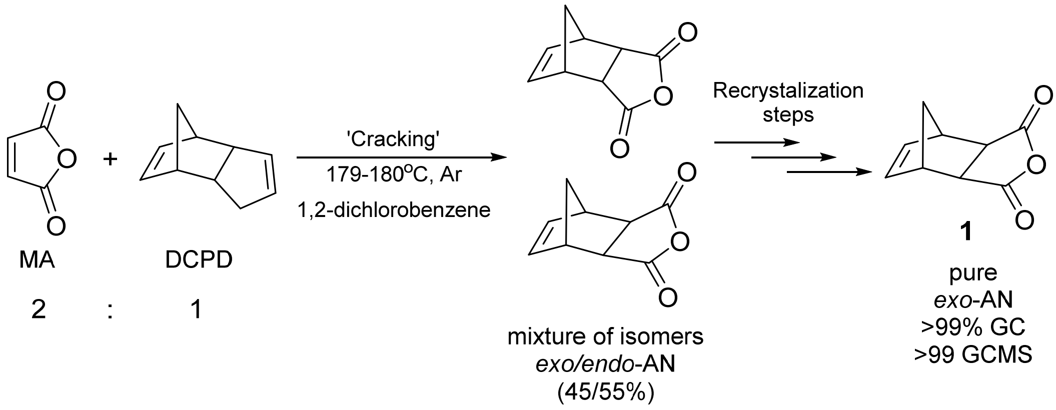

(S1)

In a three-neck round-bottom flask equipped with a magnetic stirrer, reflux condenser and dropping funnel, 122.57 g (1.25 mol) of maleic anhydride (**MA**) was weighed. Then the flask was placed in a heating mantle, the air was removed and the system was filled with argon using vacuum gas manifold (Schlenk line). 100 mL of 1,2-dichlorobenzene was added and the reactor was heated until complete dissolution of maleic anhydride. After this time, the temperature was increased to 179-180°C and 88.51 mL (0.65 mol) of dicyclopentadiene (**DCPD**) was very slowly added dropwise. After that, the reactor was left to cool overnight at room temperature. The crude product was filtered on a Bűchner funnel and washed with hexane five times to wash out residual dichlorobenzene. Next the solid (mixture of *exo-/endo*-AN) was dissolved in pure acetone and recrystallized 4 times at low temperature (4-6°C) to give white crystals of *exo*-AN (**1**) (102.5 g, 0.63 mol, yield 65%, purity >99% GC).

Analytic data: ^{1}H NMR (400 MHz, $CDCl_3$, δ (ppm)): 6.32 (s, 2H, (2 and 3)), 3.45 (s, 2H, (1 and 4)), 3.0 (s, 2H, (5 and 6), 1.66 (d, J_{HH} = 10.0Hz, 1H, (7)), 1.43 (d, J_{HH} = 10.0Hz, 1H, (7')). ^{13}C NMR (105 MHz, $CDCl_3$, δ (ppm)): 171.7 (C8 and C9), 138.1 (C2 and C3), 48.9 (C1 and C4), 47.0 (C5 and C6), 44.2 (C7). MS (EI) (m/z (relat. int. %)): $164^{•+}$ (5), 146 (2), 119 (7), 105 (2), 99 (3), 91 (88), 77 (3), 66 (100), 50 (11). HRMS (m/z) Calcd. for $C_9H_8O_3$: 164.0473, Found 164.0472. MS (FAB, m/z (%) (M+, 100): 164.0. Elemental analyses calcd. for $C_9H_8O_3$: C 65.85, H 4.91; found C 65.79 H 4.90.

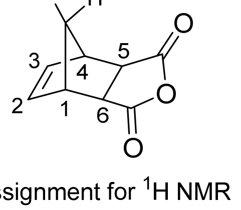

Assignment for ^{1}H NMR

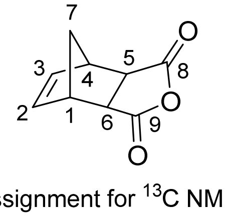

Assignment for ^{13}C NMR

***Synthesis of N-propargyl-cis-5-norbornene-exo-2,3-dicarboxylic imide* (2)**

The title imide-norbornene derivative (**2**) was prepared in accordance with a very similar methodology outlined in the literature [6] with a slight modification. In a two-neck round-bottom flask equipped with a magnetic stirrer and reflux condenser, 4.92 g (0.030 mol) of *exo*-AN (**1**). The air was removed and the system was filled with argon using vacuum gas manifold (Schlenk line). Then, 40 mL of toluene was added and the mixture was placed in an oil bath at 110°C. When, the anhydride norbornene was complete dissolution 0.284 mL (0.003 mol - calculated per amount of **1**) of acetic anhydride was added in one portion. After 10 minutes the system was cooled at 45ºC and 2.18 mL (0.034 mol) of propargylamine was added dropwise. Then the reaction system was heated at 110°C for 1 hour. The reaction progress was controlled by GC and GCMS. After full conversion of substrates, the reaction mixture was cooled at room temperature and extracted with dichloromethane and distilled water (three times) in order to remove the amine residue. The organic layer was dried over magnesium sulphate (VI) and then filtered through silica and Celite® (so-called flash column). The excess of solvent was removed in an evaporator and the final product was dried by using vacuum line system. White, small crystals were obtained in 90% yield, 5.43 g, and purity 99% GC and GCMS).

Analytic data: ^{1}H NMR (400 MHz, $CDCl_3$, δ (ppm)): 6.31 (s, 2H, (2 and 3)), 4.23 (d, J_{HH} = 3.6Hz, 2H, (8)), 3.32 (s, 2H, (1 and 4)), 2.73 (d, J_{HH} = 2.0Hz, 2H, (5 and 6)), 2.19 (s, 1H, (9)), 1.65 (d, J_{HH} = 13.2Hz, 1H, (7)), 1.43 (d, J_{HH} = 13.2Hz, 1H, (7')). ^{13}C NMR (105 MHz, $CDCl_3$, δ (ppm)): 176.8 (C8, C9), 138.1 (C2, C3), 76.6 (C12), 71.5 (C11), 48.0 (C1, C4), 45.8 (C5, C6), 43.0 (C7), 27.7 (C10). MS (EI) (m/z (relat. int. %)) $202^{\bullet+}$ (45), 184 (5), 172 (12), 136 (95), 108 (8), 91 (37), 66 (100). MS (FAB, m/z (%) (M+, 100): 201.0. Elemental Analyses Calcd. for $C_{12}H_{11}NO_2$: C 71.63, H 5.51; found C 71.61.79 H 5.50.

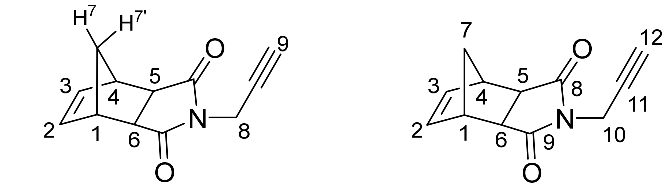

Assignment for ^{1}H NMR   Assignment for ^{13}C NMR

exo-*N*-propargyl-NBD imide

General procedure for the synthesis of new norborneno-quinine monomers 5 and 6 'Click chemistry'

The title compounds were produced according to the literature [7, 8] with appropriate modification. To a stirred solution of (8S,9S)-9-azido-(9-deoxy)dihydroepiquinine (**3**) 2,33 g (6,64 mmol, 1 eq.) and *N*-propargyl-*cis*-5-norbornene-*exo*-2,3-dicarboxylic imide (**2**) 1,33 g (6,64 mmol, 1 eq.) in mixture ethanol (30 mL) and water (15 mL), 100 mg of sodium ascorbate was added followed by 1 mL 0.5M aq. $CuSO_4$. The flask was tightly closed and the reaction was left overnight. After the consumption of substrates (TLC control) the solution was diluted with water (50 mL) and 10% aq. EDTA solution (10 mL). Mixture was extracted three times with 25 mL portions of dichloromethane. Organic phases were combined and dried over anhydrous Na_2SO_4. The solvent was then evaporated under reduced pressure to give crude product, which after column chromatography on silica gel with the use of **2** to 5% MeOH/dichloromethane as mobile phase gave pure monomer as pale yellow oil (2,58 g, 70%). Monomer **5** can be obtained in the solid state by dissolving the product in hot AcOEt, followed by precipitation with excess hexane at low temperature and drying using a vacuum pump system.

*[N-[(8S,9S)-9-((1,2,3-triazole4-yl)methyl)-(9-deoxy)dihydroepiquinine]-cis-5-norbornene-exo-2,3-dicarboximide] (**5**)*

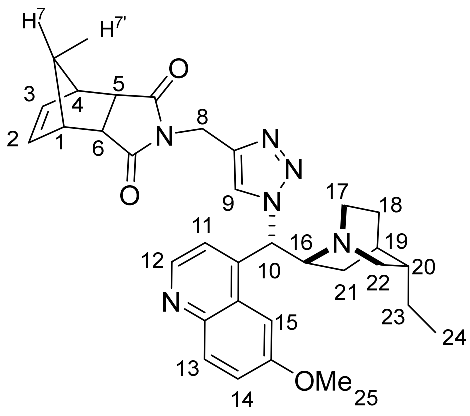

Analytic data: ^{1}H NMR (400 MHz, $CDCl_3$, δ (ppm)): 8.80 (d, J_{HH} = 4.8Hz, 1H, (12)), 8.01 (d, J_{HH} = 9.2Hz,1H, (13)), 7.52 (s, 1H, (9)), 7.48 (d, J_{HH} = 2.8Hz, 1H), 7.46 (d, J_{HH} = 4.4Hz, 1H, (14)), 7.38 (d, J_{HH} = 2.4Hz, 1H, (15)), 7.36 (d, J_{HH} = 2.4Hz, 1H, (11)), 6,42 (d, J_{HH} = 10.8Hz, 1H, (10)), 6.22 (s, 2H, (2 and 3)), 4.67 (d, J_{HH} = 1.6Hz, 2H, (8)), 3.95 (s, 3H, (25)), 3.39 (s, br, 1H,(16)), 3.19 (s, 1H), 3.14 (s, 2H, (1 and 4)), 2.71 (t, J_{HH} = 10.0Hz, 12.0Hz, 2H, (17)), 2.62 (d, J_{HH} = 1.2Hz, 2H, (5 and 6)), 2.47 (d, J_{HH} = 12.8Hz, 2H, (22)), 1.84 (t, 2H, (21)), 1.74 (s, 1H, (19)), 1.61 (m, 1H, (20)), 1.52 (m, 2H, (18)), 1.43 (q, J_{HH} = 10.0Hz, 2H, (23)), 1.24 (d, J_{HH} = 10.0Hz, 1H, (7)), 0.94 (d, J_{HH} = 10.0Hz, 1H, (7')), 0.89 (t, J_{HH} = 7.2Hz, 7.2Hz, 3H, (24)). ^{13}C NMR (105 MHz, $CDCl_3$, δ (ppm)): 177.4, 177.3, 158.8, 147.5, 145.1, 141.9, 139.4, 138.0, 132.1, 128.3, 122.5, 121.9, 119.5, 100.9, 58.0, 55.9, 47.9, 45.4, 42.6, 41.2, 37.3, 33.8, 28.6, 27.9, 27.4, 25.6, 12.2. MS (FAB, m/z (%) (M+, 100): 552.3. Elemental Analyses Calcd. for $C_{32}H_{36}N_6O_3$: C 69.54, H 6.57; found C 68.82 H 6.54. Melting point, m.p. 184-185°C.

Monomer **6** was synthesised in a similar manner to compound **5** using [(8S,9S)-9-azido-(9-deoxy)-epiquinine] (**4**) 0,68 g (1,94 mmol, 1 eq.) and *N*-propargyl-*cis*-5-norbornene-*exo*-2,3-dicarboximide (**2**) 0,39 g (1,94 mmol, 1 eq.) dissolved in a mixture EtOH/water (12+8 mL). Monomer **6** can be obtained in the solid state by precipitation from DCM with excess hexane and drying using a vacuum pump system. The final product was isolated as a light beige solid whit 0,93 g (86% yield).

*[N-[(8S,9S)-9-((1,2,3-triazol-4-yl)methyl)-(9-deoxy)-epiquinine]-cis-5-norbornene-exo-2,3-dicarbox-imide] **(6)***

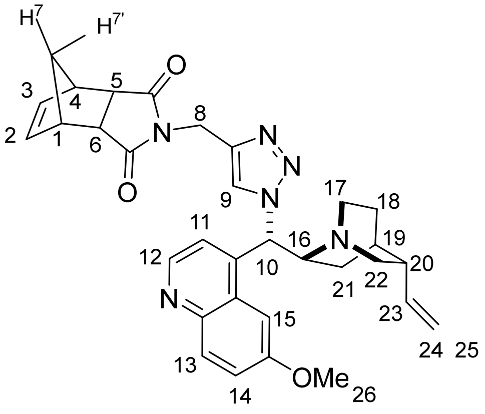

Analytic data: ^{1}H NMR (400 MHz, $CDCl_3$, δ (ppm)): 8.80 (d, J_{HH} = 6.0Hz, 1H, (12)), 8.01 (d, J_{HH} = 12.0Hz,1H, (13)), 7.49 (s, 1H, (9)), 7.46 (d, J_{HH} = 3.6Hz, 1H), 7.45 (d, J_{HH} = 6.4Hz,, 1H, (14)), 7.37 (d, J_{HH} = 3.6Hz, 1H, (15)), 7.35 (d, J_{HH} = 3.6Hz, 1H, (11)), 6,37 (d, J_{HH} = 15.2Hz, 1H, (10)), 6.22 (s, 2H, (2 and 3)), 5.90 (dd, J_{HH} = 9.2Hz, 6.8Hz, J_{HH} = 6.8Hz, 9.6Hz, 1H, (23)), 5.12 (d, J_{HH} = 1.6Hz, 1H, (24)), 5.07 (d, J_{HH} = 9.6Hz, 1H, (25)), 4.66 (d, J_{HH} = 2.0Hz, 2H, (8)), 3.93 (s, 3H, (26)), 3.73 (t, J_{HH} = 10.0Hz, J_{HH} = 7.6Hz, 1H,(16)), 3.35 (m, 1H), 3.19 (s, 1H), 3.15 (s, 2H, (1 and 4)), 3.05 (m, 1H, (22)), 2.72 (m, 1H, (22)), 2.70 (m, 1H (17)), 2.62 (d, J_{HH} = 1,2Hz, 2H, (5 and 6)), 2.33 (m, 1H, (20)), 1.90 (m, 2H, (21)), 1.78 (s, 1H, (19)), 1.60 (t, J_{HH} = 8.8Hz, J_{HH} = 7.6Hz, 2H, (18)), 1.26 (trace of silicon grease), 1.21 (d, J_{HH} = 13.6Hz, 1H, (7)), 0.90 (d, J_{HH} = 13.2Hz, 1H, (7')), 0.88 (trace of silicon grease). ^{13}C NMR (105 MHz, $CDCl_3$, δ (ppm)): 177.3, 177.1, 158.6, 147.3, 145.0, 141.9, 141.4, 139.0, 137.9, 137.8, 132.0, 128.1, 122.4, 121.7, 119.4, 114.8, 100.7, 67.9, 57.9, 56.0, 55.8, 47.7, 45.3, 42.4, 41.0, 39.2, 33.6, 27.7, 27.6, 27.5. MS (FAB, m/z (%) (M+, 100): 550.2. Elemental Analyses Calcd. for $C_{32}H_{34}N_6O_3$: C 69.80, H 6.22; found C 68.54 H 6.20. Melting point: m.p. 205-208°C.

General procedure for self-metathesis of quinine

In a two-neck small glass reactor (5 ml) equipped with a magnetic stirrer and a reflux condenser, 0.1 g ($3.09x10^{-4}$ mole) of quinine (QN) was weighed and dissolved in 1.23 mL of DCM (0.25 M). Subsequently, $6.17x10^{-6}$ mol or $3.08x10^{-6}$ mol of Grubbs catalysts G1 or G2 in DCM were added to the solution with the substrate. The molar ratio of alkaloid to catalyst was 50:1 or 100:1, as observed in the polymerization reactions of monomers **5** and **6**. The system was subsequently subjected to testing at ambient temperature (25°C), as well as elevated temperatures of 30°C and 40°C. Subsequent to an elapsed time of 3-4 hours, a sample was extracted and the surplus solvent was evaporated. Thereafter, ^{1}H NMR studies were conducted. A detailed analysis of the spectra indicated an absence of formation of the self-coupling product (Figure S25 and Figure S26).

Poly(*exo*-N-((1,2,3-triazole-4-yl)methyl)-(9-deoxy)dihydroepiquinine))norbornene-5,6-dicarboxy imide)s - P1

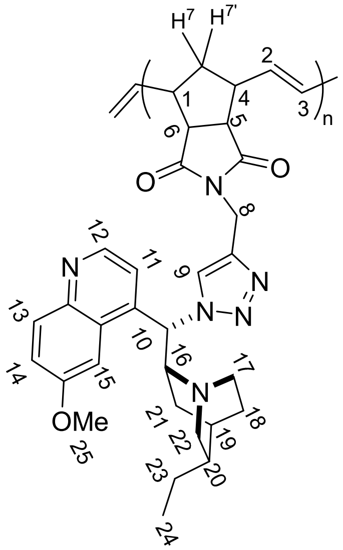

Analytic data: ^{1}H NMR (400 MHz, $CDCl_3$, δ (ppm)): 8.77 (d, J_{HH} = 16.4Hz, 1H, (12)), 8.01 (d, J_{HH} = 9.2Hz,1H, (13)), 7.67 (s, br, 1H, (9)), 7.49 (d, J_{HH} = 2.8Hz, 1H, (14)), 7.33 (d, J_{HH} = 14.0Hz, 1H, (15)), 7.25 (d, J_{HH} = 2.4Hz, 1H, (11)), 6,40 (s, br, 1H, (10)), 5.65-5.58 (s, br, 2H, E- >*H*C=C*H*< (2 and 3)), 5.44 (s, br, 2H, Z- >*H*C=C*H*< (2 and 3)), 4.62 (s, br, 2H, (8)), 3.92 (d, J_{HH} = 11,2Hz, 3H, (25)), 3.39 (s, br, 1H,(16)), 3.14 (s, br, 2H, (1 and 4)), 2.94 (s, br, 2H, (17)), 2.68 (s, br, 2H, (5 and 6)), 2.46 (s, br, 2H, (22)), 2.17 (s, br, 2H, (21)), 1.95 (s, 1H, (19)), 1.69 (1, 1H, (20)), 1.62 (s, br, 2H, (18)), 1.42-1.39 (q,, br, J_{HH} = 7.6Hz, 2H, (23)), 1.25 (s, 1H, (7)), 0.88 (s, 1H, (7')), 0.84 (s, br, 3H, (24)). ^{13}C NMR (105 MHz, $CDCl_3$, δ (ppm)): 177.7, 158.8, 147.7, 145.1, 142.2, 139.4, 132.2, 128.3, 122.5, 119.9, 101.0, 58.1, 58.0, 56.0, 51.0, 45.7, 41.3, 37,2, 35.6, 33.9, 31.1, 29.9, 28.4, 27.7, 26.8, 25.6, 12.3. Elemental Analyses Calcd. for mer $C_{33}H_{37}N_6O_3$: C 70.07, H 6.59; found C 69.54 H 6.57.

Poly(*exo*-N-((1,2,3-triazole-4-yl)methyl)-(9-deoxy)epiquinine))norbornene-5,6-dicarboxyimide)s-P2

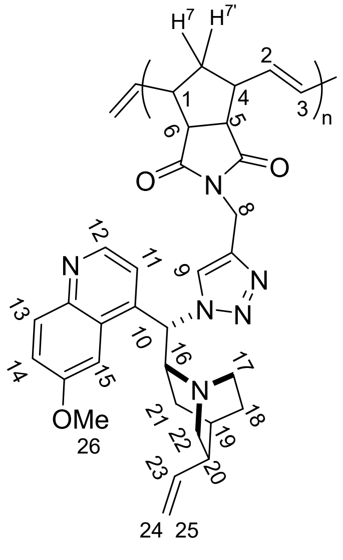

Analytic data: ^{1}H NMR (400 MHz, $CDCl_3$, δ (ppm)): 8.76 (s, br, 1H, (12)), 7.99 (s, br, 1H, (13)), 7.70 (d, J_{HH} = 3.6Hz, 1H), 7.52 (d, J_{HH} = 6.4Hz,, 1H, (14)), 7.46 (s, br, 1H, (15)), 7.35 (s, br, 1H, (11)), 6,36 (s, br, 1H, (10)), 5.85 (s, br, 1H, (23)), 5.58 (s, 2H, E- >HC=CH<(2 and 3)), 5.36 (s, br, 2H, Z- >HC=CH< (2 and 3)), 5.29 (DCM solvent), 5.09-5.04 (m, br, 2H, (24 and 25)), 4.64 (d, J_{HH} = 12.0Hz, 2H, (8)), 4.20 (solvent), 3.95 (s, br, 1H,(16)), 3.93 (s, br, 3H, (26)), 3.14 (s, br, 2H, (1 and 4)), 2.92 (s,

br, 1H, (22)), 2.69 (s, br, 2H, (22 and 17)), 2.62 (s, br, 2H, (5 and 6)), 2.26 (m, 1H, (20)), 1.92-1.88 (m, br, 2H, (21)), 1.78 (s, br, 1H, (19)), 1.59 (s, br, 2H, (18)), 1.26 (trace of silicon grease), 1.31 (s, br, 1H, (7)), 0.93 (d, J_{HH} = 13.2Hz, 1H, (7')), 0.88 (trace of silicon grease). ^{13}C NMR (105 MHz, $CDCl_3$, δ (ppm)): 177.4, 158.6, 147.4, 144.9, 142.0, 141.7, 139.1, 138.5, 134.8, 132.0, 130.9, 128.8, 128.1, 122.3, 119.5, 115.7, 114.9, 100.8, 68.1, 60.4, 58.0, 55.8, 50.6, 47.1, 46.6, 42.8, 41.0, 39.1, 38.7, 33.6, 30.9, 30.3, 29.6, 28.9, 27.6, 23.7, 23.0, 14.0, 10.9. Elemental Analyses Calcd. for mer $C_{33}H_{35}N_6O_3$: C 70.32, H 6.26; found C 68.89 H 6.18.

NMR Spectra

Compound 1

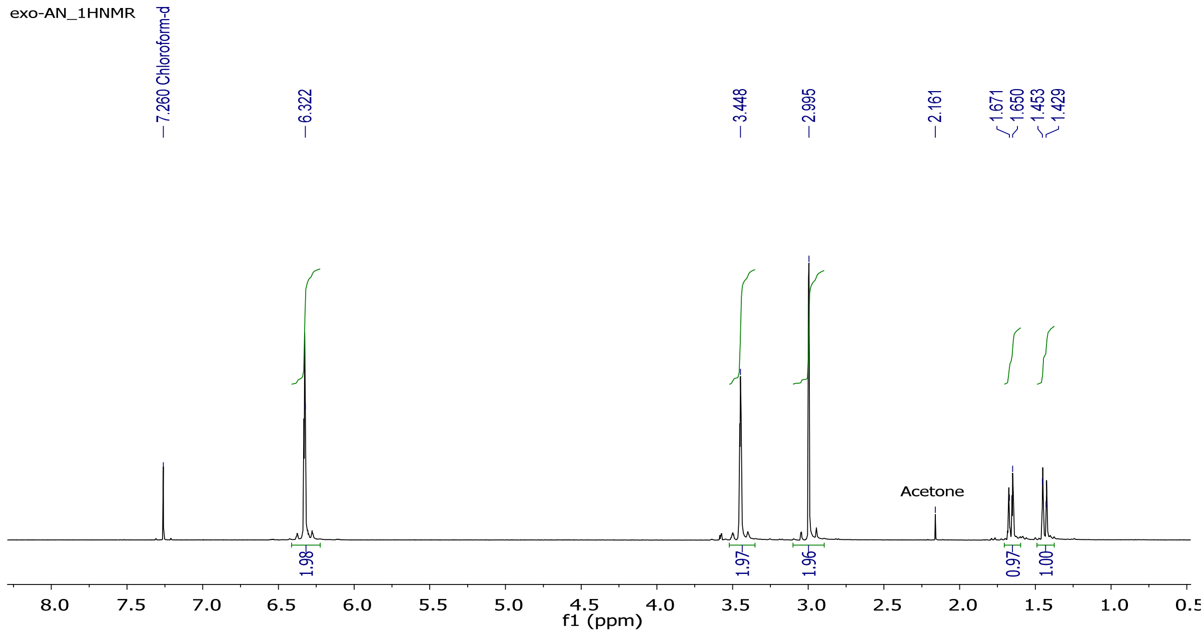

Figure S1. ^{1}H NMR spectrum of compound **1** in $CDCl_3$ at 25°C.

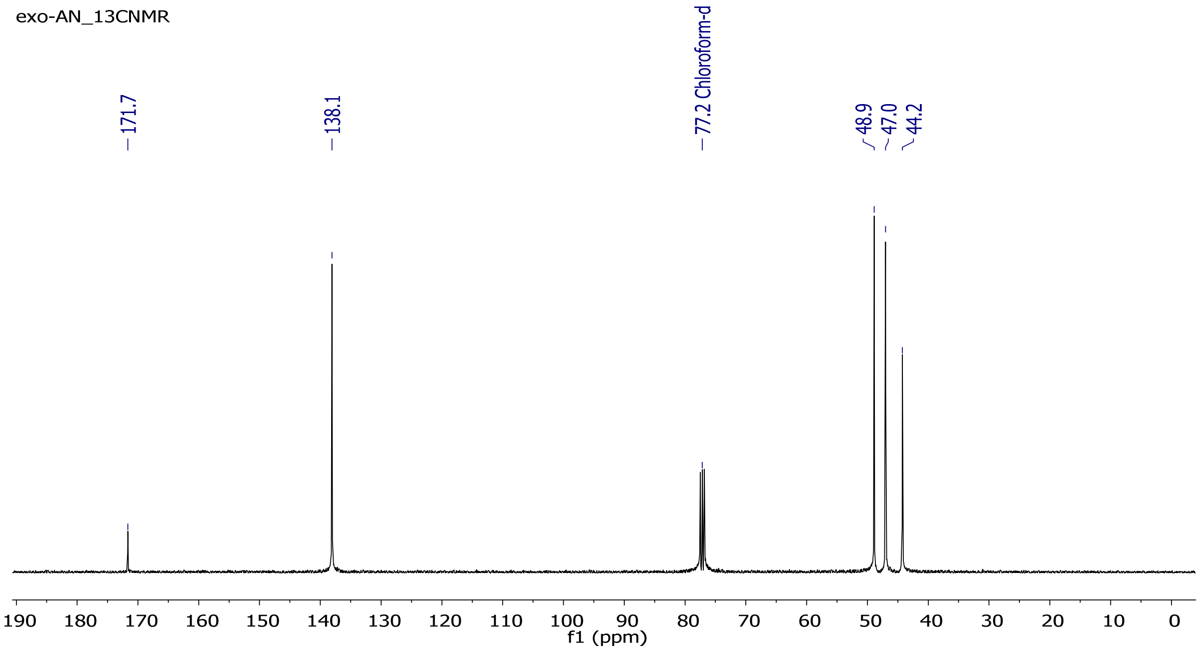

Figure S2. ^{13}C NMR spectrum of compound **1** in $CDCl_3$ at 25°C.

Compound 2

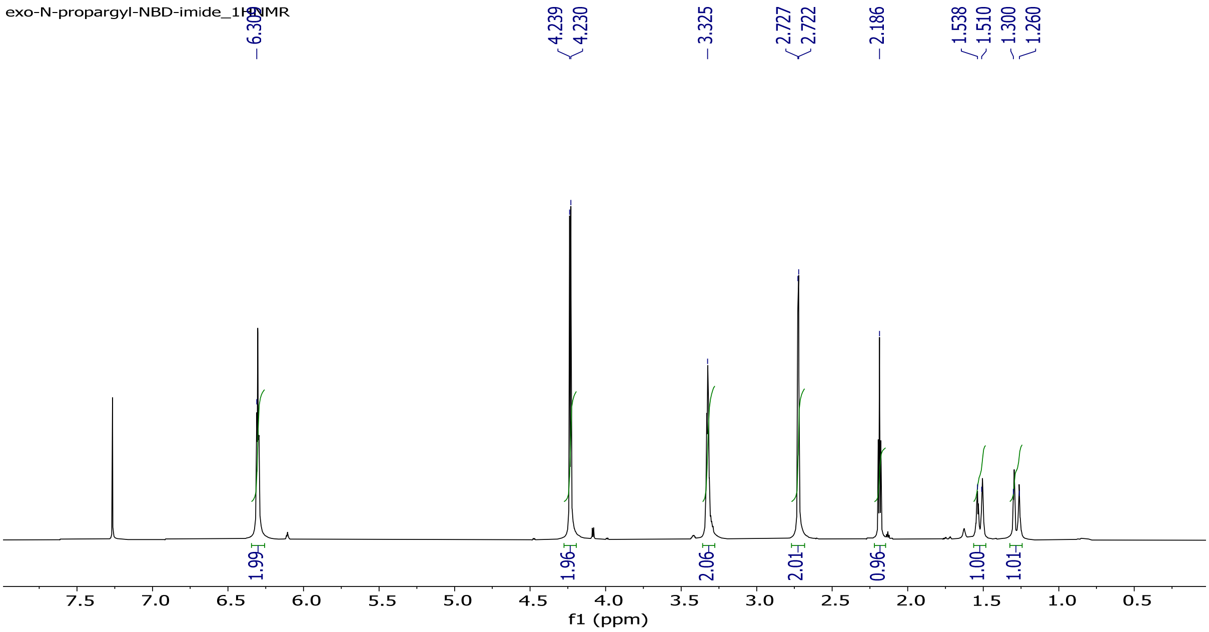

Figure S3. ^{1}H NMR spectrum of compound **2** in $CDCl_3$ at 25°C.

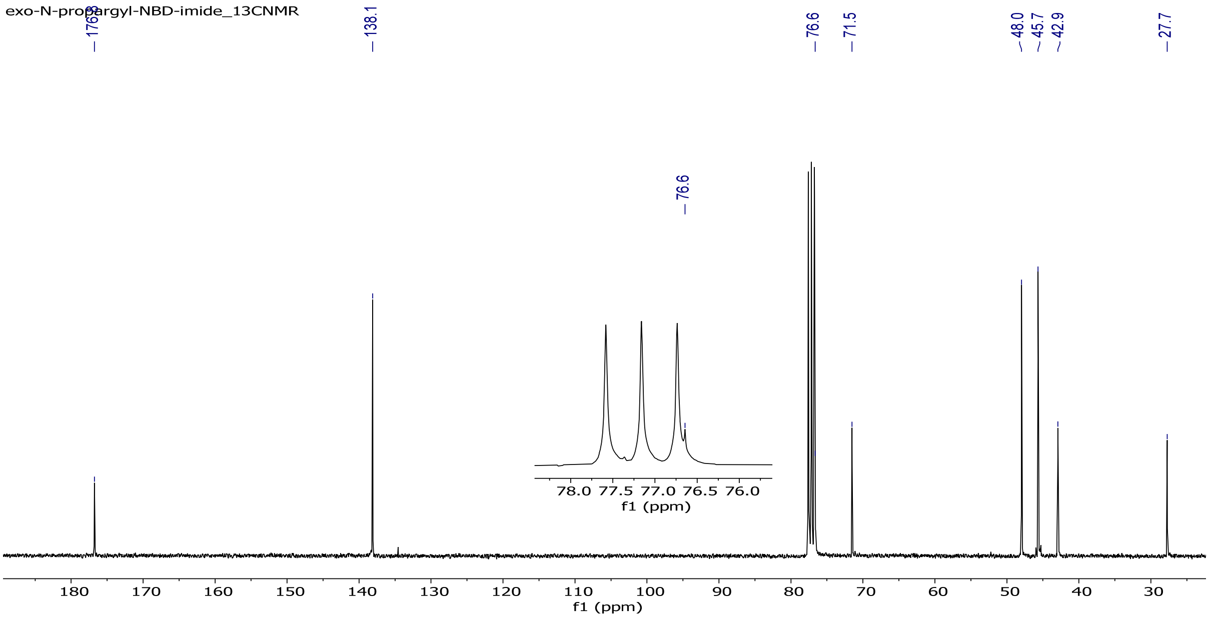

Figure S4. ^{13}C NMR spectrum of compound **2** in $CDCl_3$ at 25°C.

Monomer 5

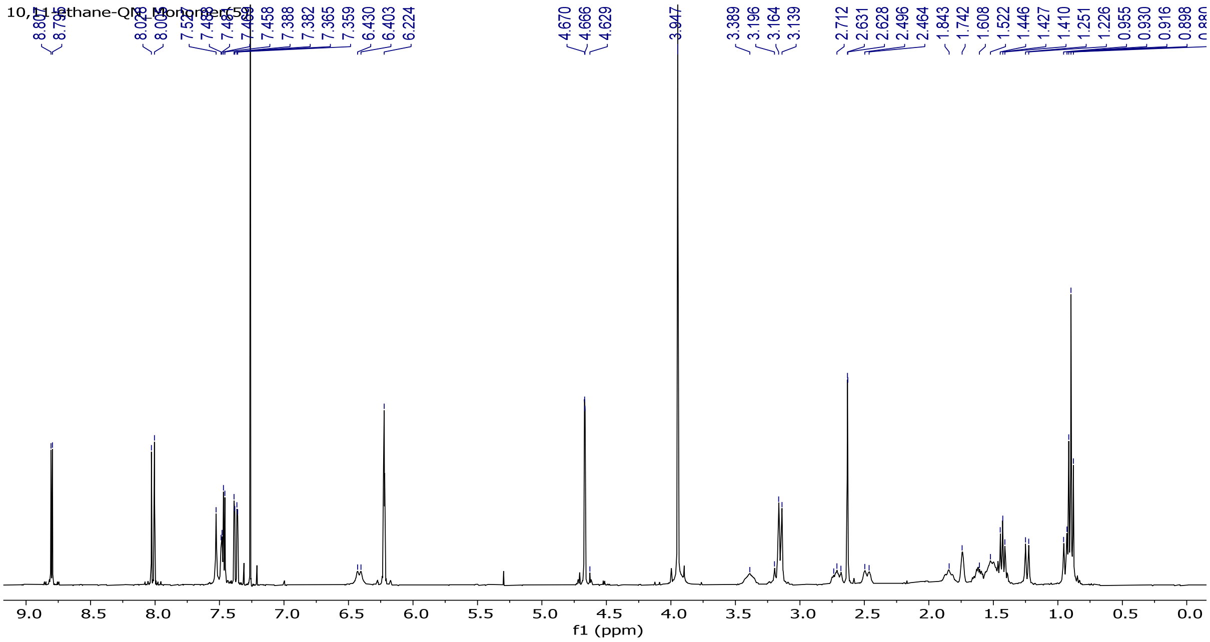

Figure S5. ^{1}H NMR spectrum of compound **5** in $CDCl_3$ at 25°C.

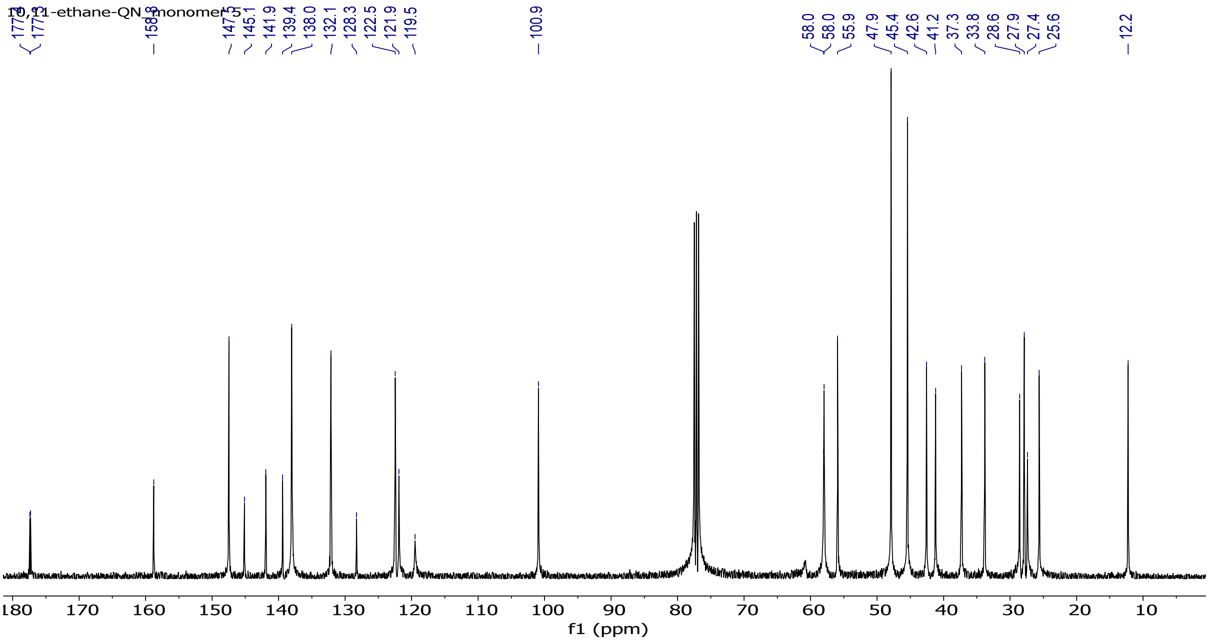

Figure S6. ^{13}C NMR spectrum of compound **5** in $CDCl_3$ at 25°C.

Monomer 6

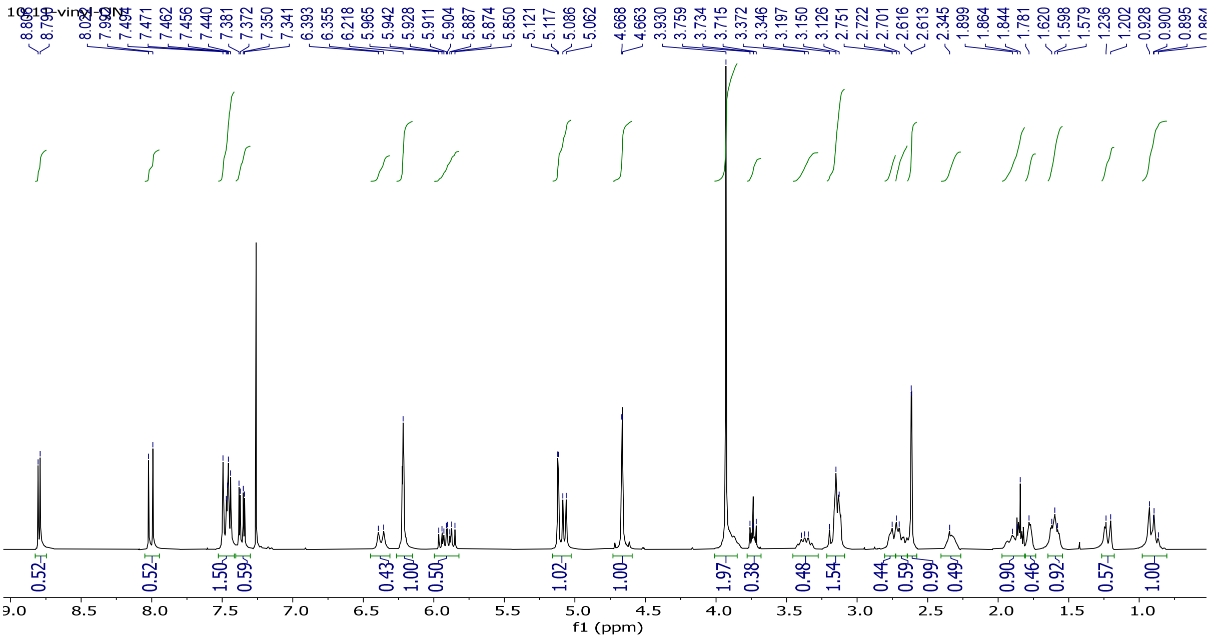

Figure S7. ^{1}H NMR spectrum of compound **6** in $CDCl_3$ at 25°C.

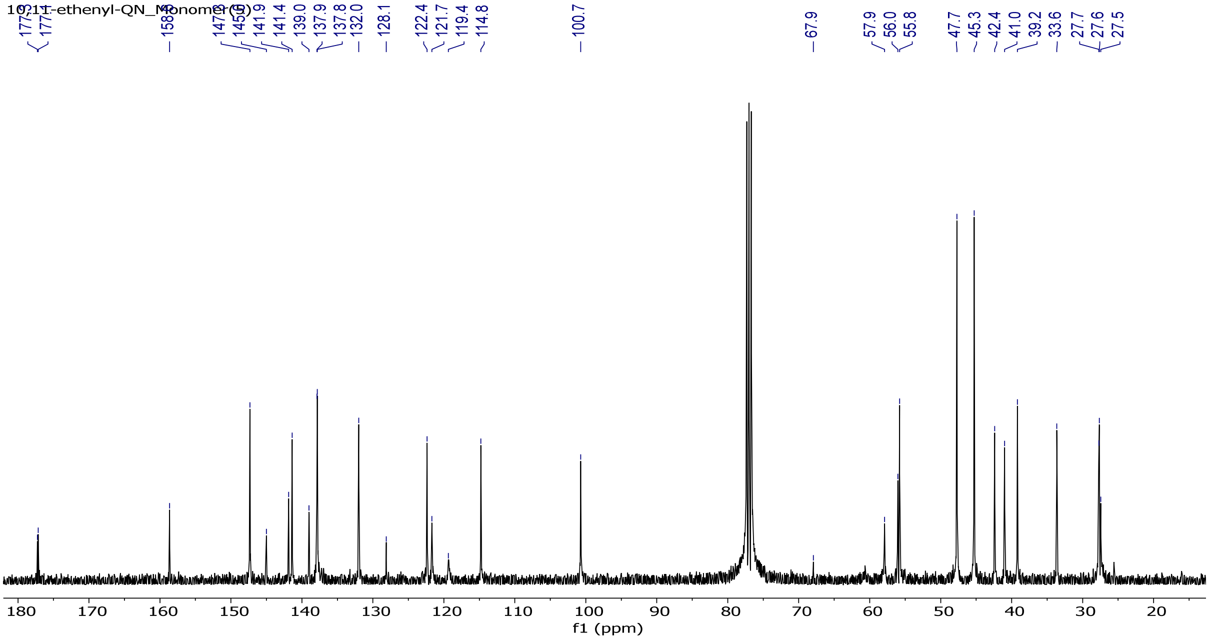

Figure S8. ^{13}C NMR spectrum of compound **6** in $CDCl_3$ at 25°C.

Polymers P1, P2 ^{1}H and ^{13}C NMR spectra

Polymer P1

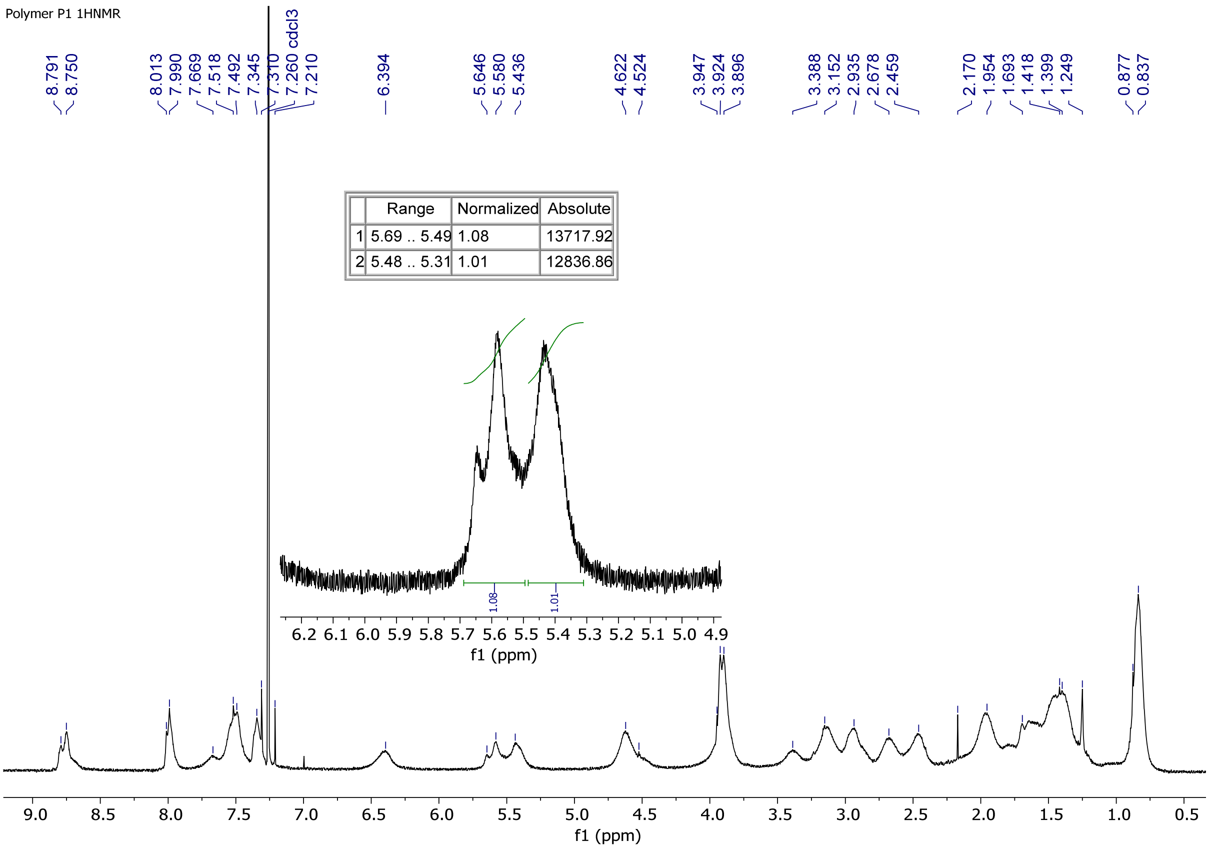

Figure S9. ^{1}H NMR spectrum of compound **P1** in $CDCl_3$ at 25°C.

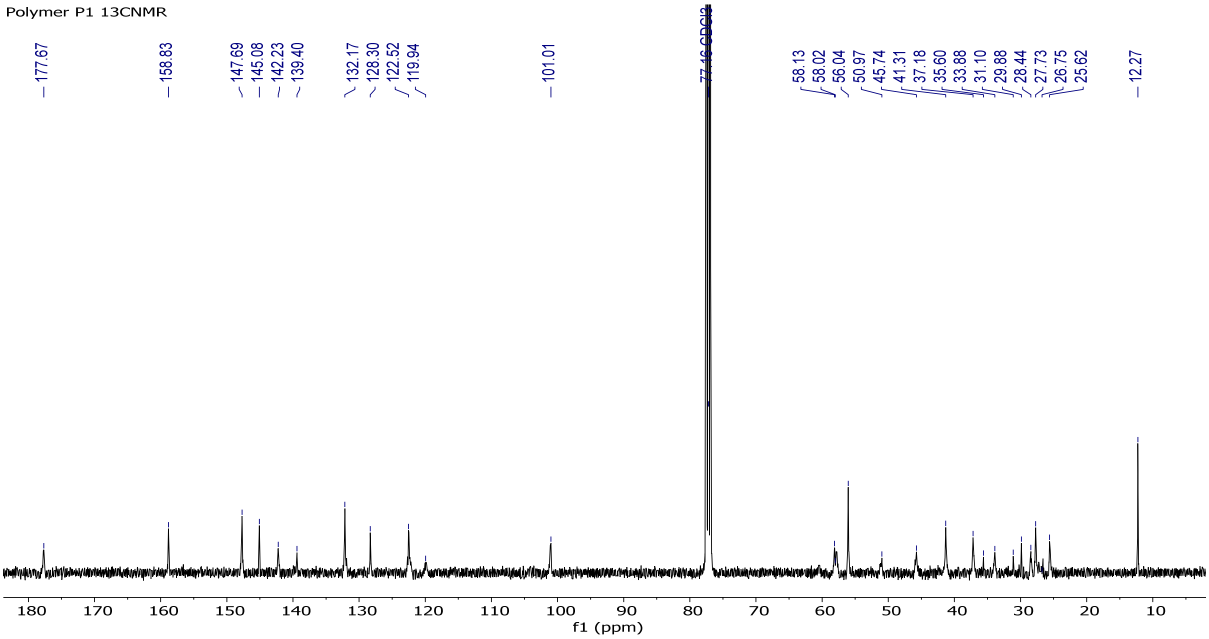

Figure S10. ^{13}C NMR spectrum of compound **P1** in $CDCl_3$ at 25°C.

Polymer P2

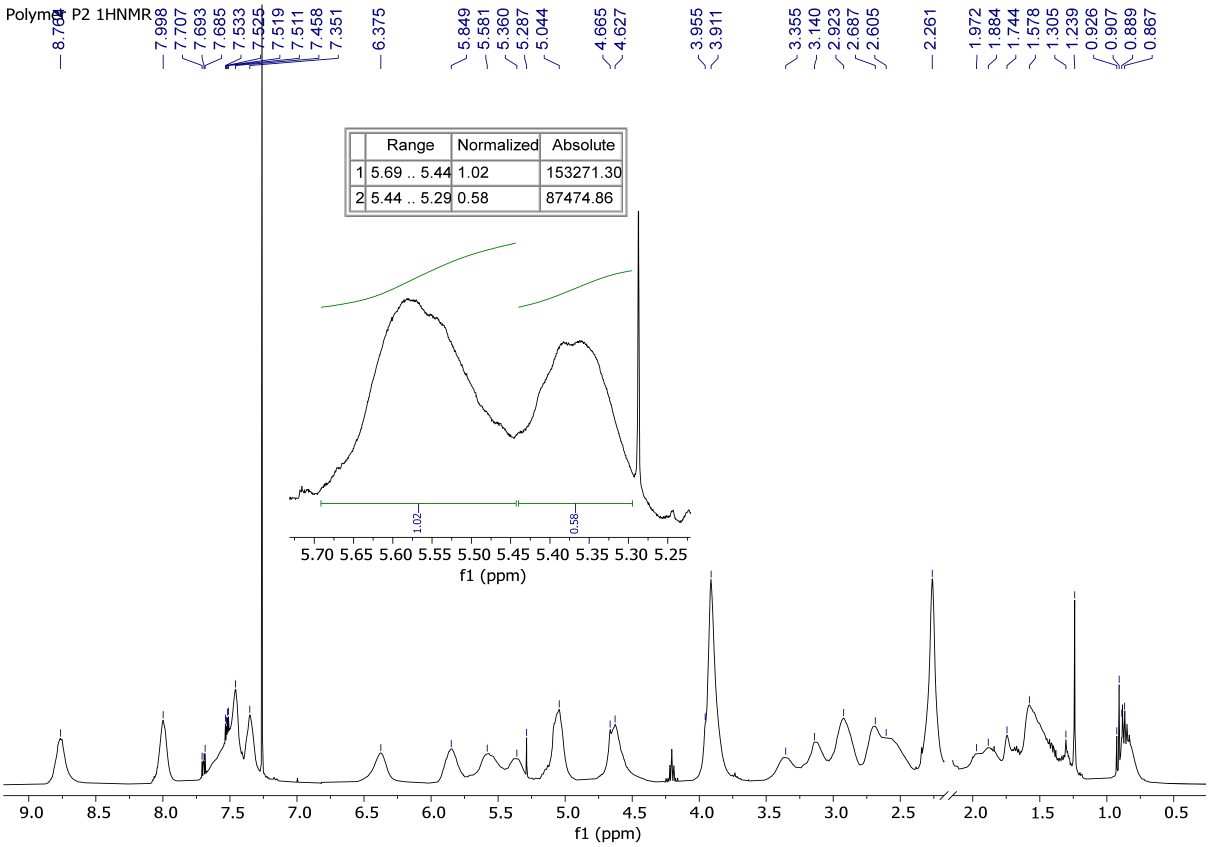

Figure S11. ^{1}H NMR spectrum of compound **P2** in $CDCl_3$ at 25°C.

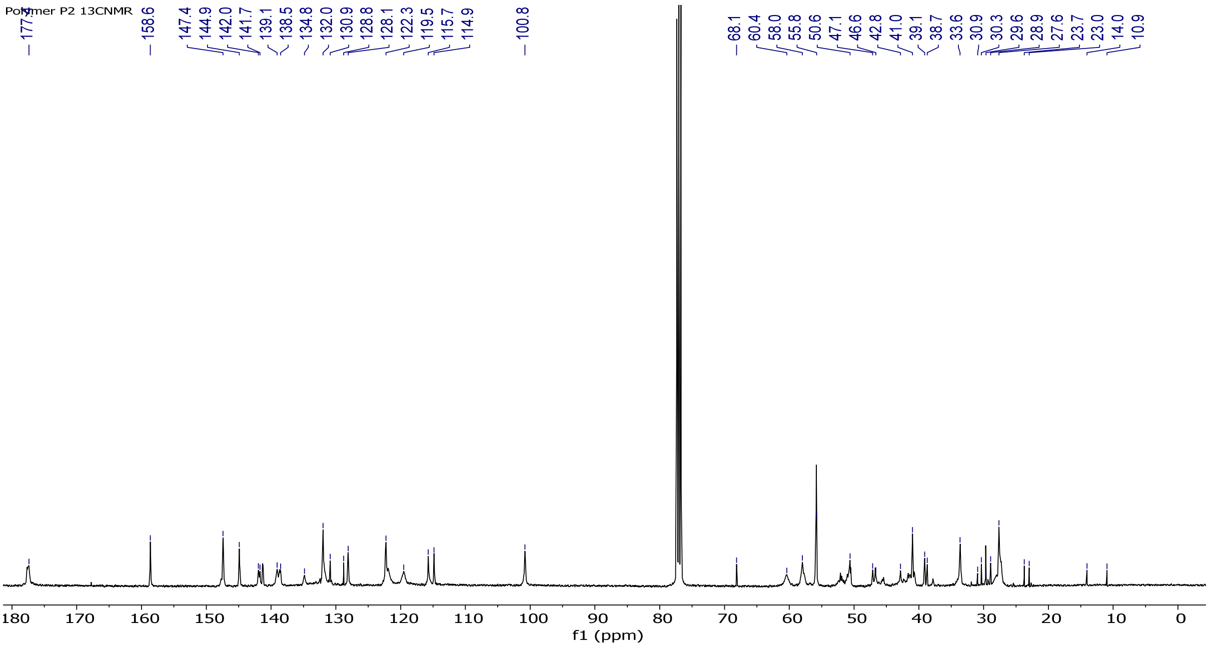

Figure S12. ^{13}C NMR spectrum of compound **P2** in $CDCl_3$ at 25°C.

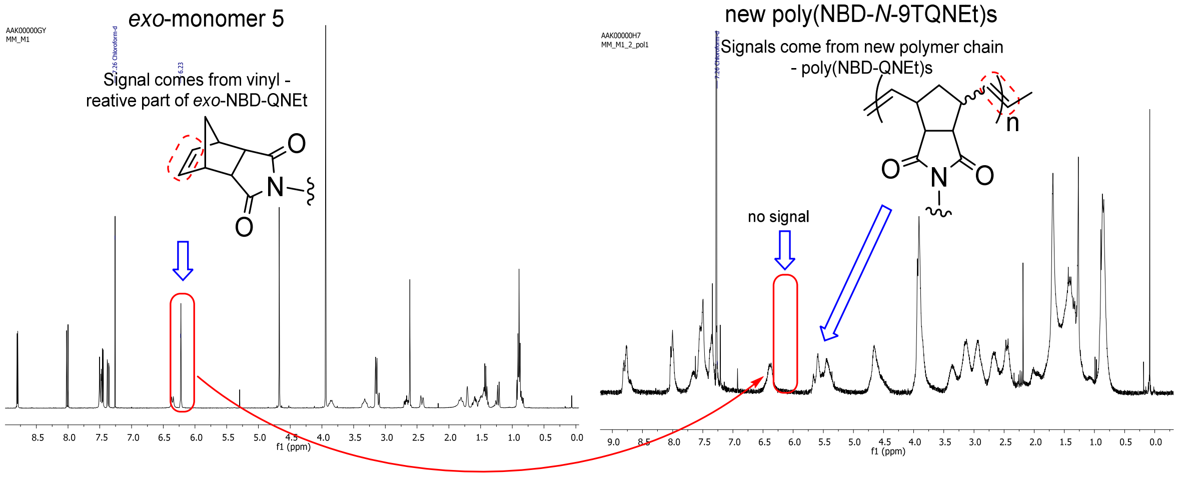

Figure S13. Comparison of two ^{1}H NMR spectra of monomer **5** and polymer **P1** taken in $CDCl_3$ at 25°C.

Thermogravimetric analysis (TGA)

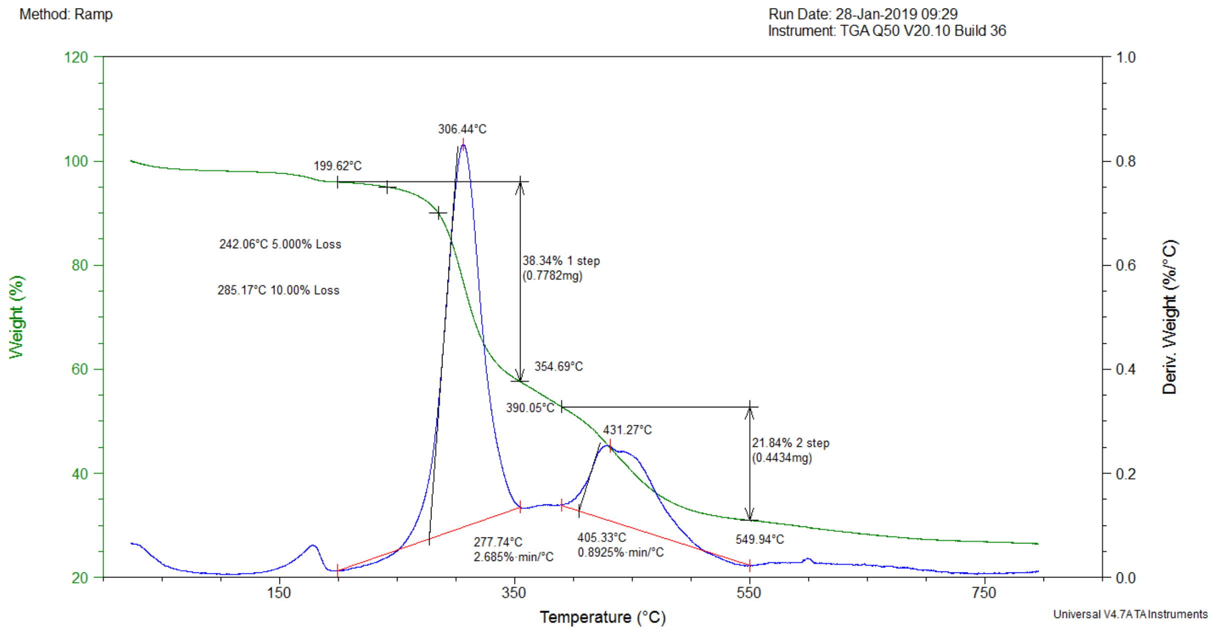

Figure S14. TGA analysis of polymer **P1(G1)** (1 mol%)

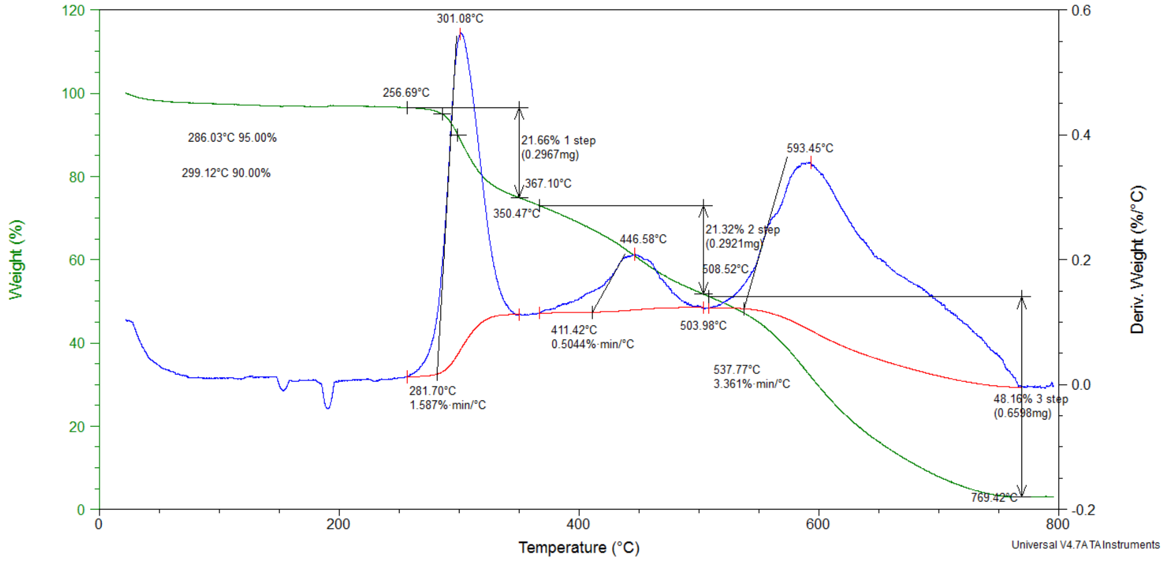

Figure S15. TGA analysis of polymer **P1(G2)** (1 mol%)

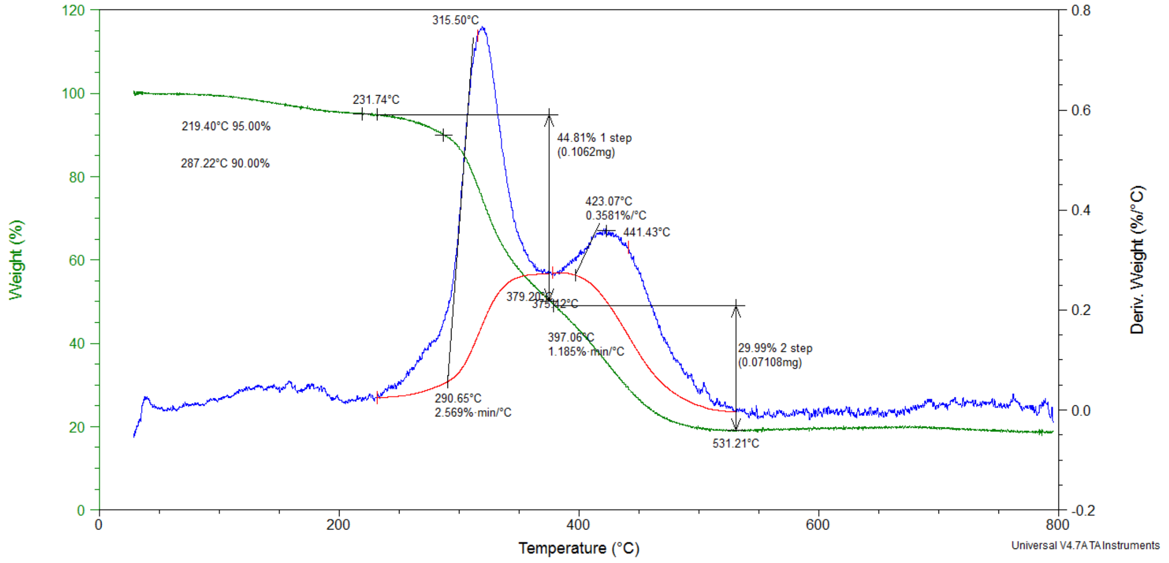

Figure S16. TGA analysis of polymer **P2(G1)** (1 mol%)

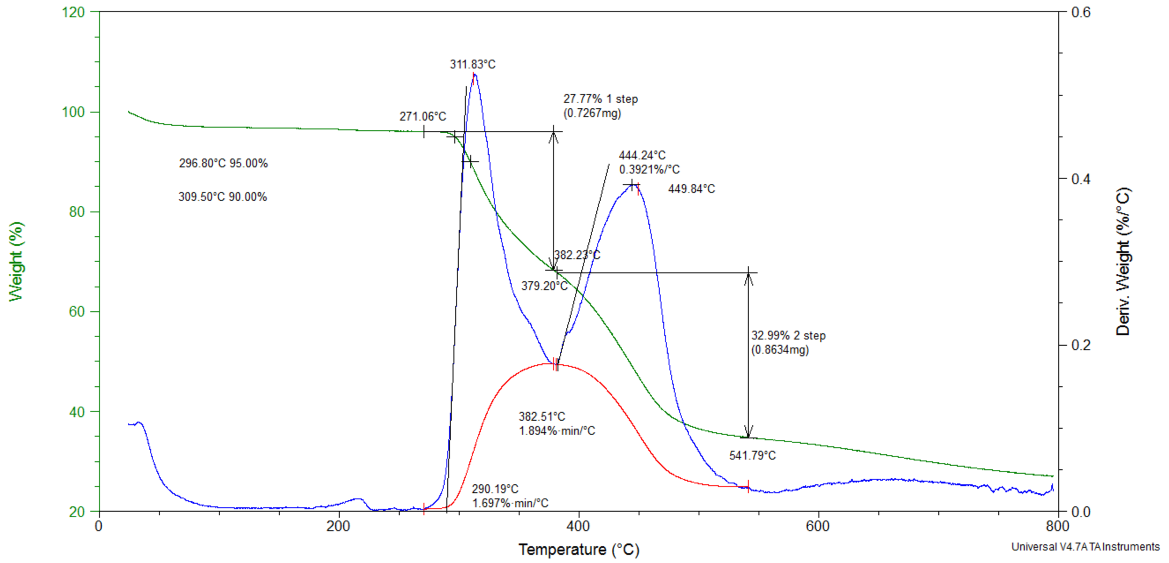

Figure S17. TGA analysis of polymer **P2(G2)** (1 mol%)

Gel permeation chromatography (GPC) analysis

Polymer **P1**

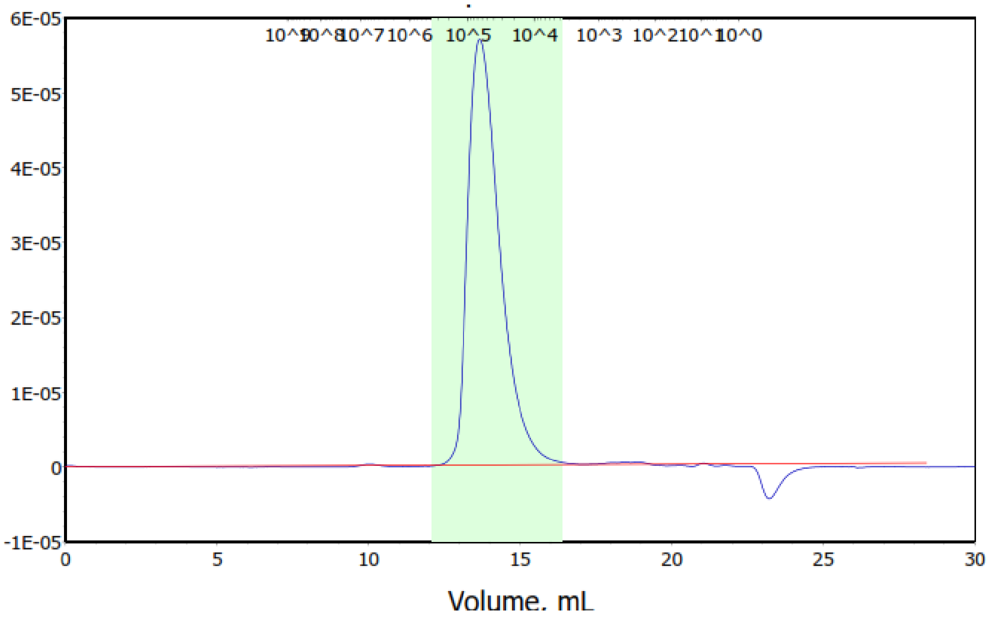

Figure S18. GPC chromatogram and data of polymer **P1**

Table S1. GPC parameters analysis of P1.

Comments: Peak 1	Calibration Details:	Function:
Left (mL): 12.09	Column Set: 2XPLGel5 microns MIXED-C	a[3]*V^3+a[2]*V^2+a[1]*V+a[0]
Right (mL): 16.37	Standards: polystyrene	a[0]= 46.649331
Mn: 44,073	Solvent: methylene chloride	a[1]= -5.3580971
Mw: 58,039	Temperature: 30°C	a[2]= 0.28821139
Mp: 64,900	Flow Rate: 0.8 mL/min	a[3]= -0.006400457
Mw/Mn: 1.317	Delay Volume: 0.155 mL	r^2= 0.99718092
Area: 0.0054096	Reference Vol.: 20.54	
Fraction: 1.0000	Operator: BW	

Polymer **P2**

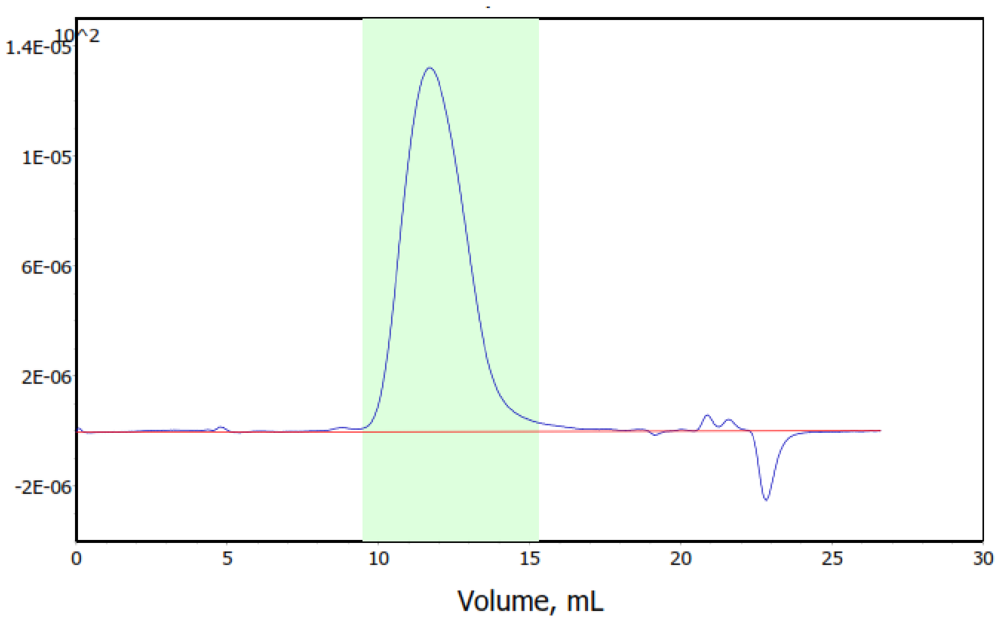

Figure S19. GPC chromatogram and data of polymeric material **P2**

Table S2. GPC parameters analysis of P2.

Comments: Peak 1	Calibration Details:	Function:
Left (mL): 12.53	Column Set: 2XPLGel5 microns MIXED-C	a[3]*V^3+a[2]*V^2+a[1]*V+a[0]
Right (mL): 15.76	Standards: polystyrene	a[0]= 46.649331
Mn: 45,235	Solvent: methylene chloride	a[1]= -5.3580971
Mw: 60,988	Temperature: 30°C	a[2]= 0.28821139
Mp: 81,868	Flow Rate: 0.8 mL/min	a[3]= -0.006400457
Mw/Mn: 1.348	Delay Volume: 0.155 mL	r^2= 0.99718092
Area: 0.0060744	Reference Vol.: 20.54	
Fraction: 1.0000	Operator: BW	

Differential Scanning Calorimetry (DSC) spectra and data of P1 and P2

DSC thermochromatogram and data of polymer P1

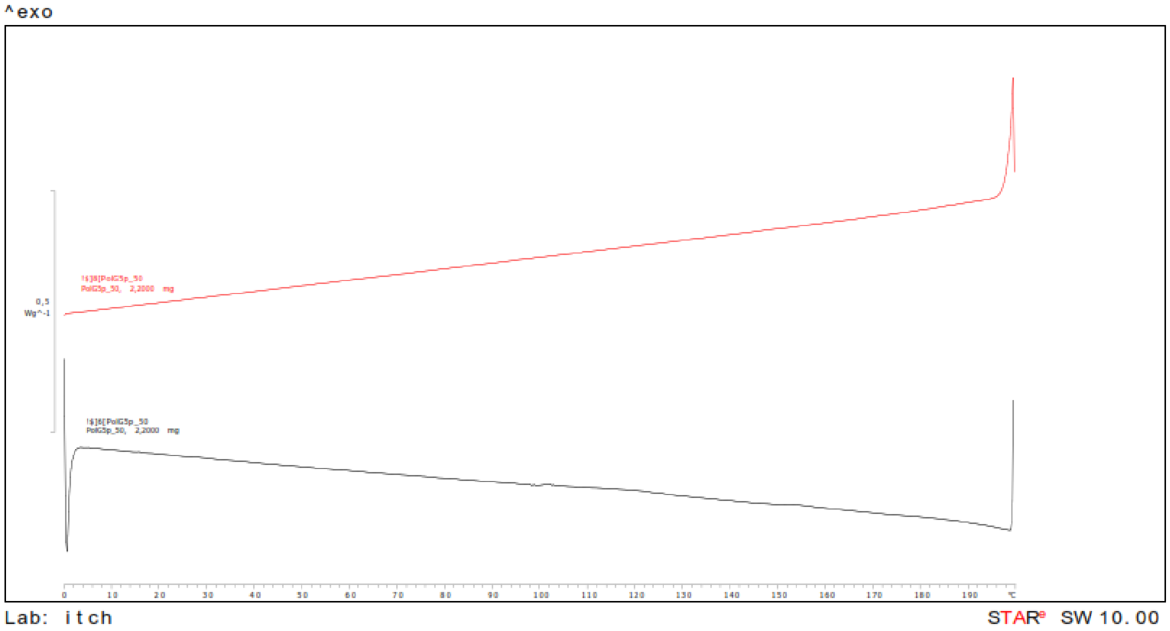

Figure S20. DSC chromatogram of polymer **P1** [mon.]:[cat.] = 100:1.

Curve: PolG5p_50
Sample: PolymerP1(G5p_50), 2,2000 mg
Module: DSC1/700/1450 GC10, 19.07.2012 12:17:31
Sample Holder: Aluminum Standard 40ul
Weight : 49.88
Material: Aluminium
Method: bodo_0_200_35mL
dt 1,00 s
[1] 0,0 °C, 10,00 min N2 35,0 ml/min
[2] 0,0..200,0 °C, 10,00 K/min N2 35,0 ml/min
[3] 200,0 °C, 5,00 min N2 35,0 ml/min
[4] 200,0..0,0 °C, -10,00 K/min N2 35,0 ml/min
[5] 0,0 °C, 5,00 min N2 35,0 ml/min
[6] 0,0..200,0 °C, 10,00 K/min N2 35,0 ml/min
[7] 200,0 °C, 5,00 min N2 35,0 ml/min
[8] 200,0..0,0 °C, -10,00 K/min N2 35,0 ml/min
[9] 0,0 °C, 5,00 min N2 35,0 ml/min
Synchronization enabled

DSC thermochromatogram and data of polymer P2

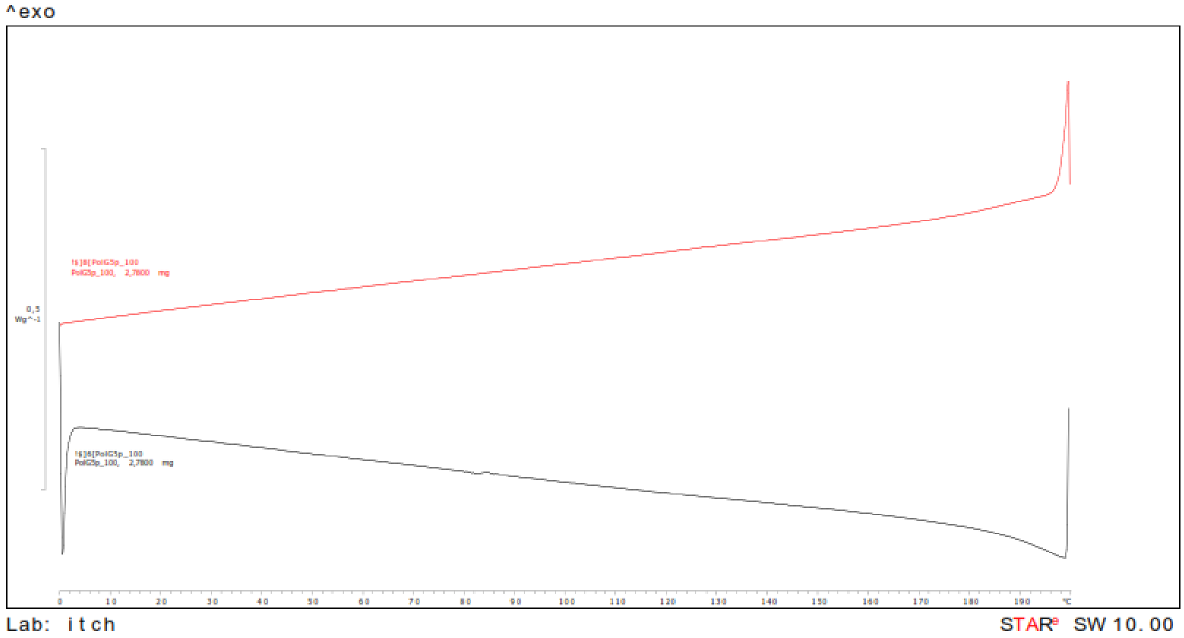

Figure S21. DSC chromatogram of polymer **P2** – [mon.]:[cat.] = 100:1.

Curve: PolG5p_100
Sample: PolymerP2(G5p_100), 2,7800 mg
Module: DSC1/700/1450 GC10, 19.07.2012 12:17:31
Sample Holder: Aluminum Standard 40ul
Weight : 49.8
Material: Aluminium
Method: bodo_0_200_35mL
dt 1,00 s
[1] 0,0 °C, 10,00 min N2 35,0 ml/min
[2] 0,0..200,0 °C, 10,00 K/min N2 35,0 ml/min
[3] 200,0 °C, 5,00 min N2 35,0 ml/min
[4] 200,0..0,0 °C, -10,00 K/min N2 35,0 ml/min
[5] 0,0 °C, 5,00 min N2 35,0 ml/min
[6] 0,0..200,0 °C, 10,00 K/min N2 35,0 ml/min
[7] 200,0 °C, 5,00 min N2 35,0 ml/min
[8] 200,0..0,0 °C, -10,00 K/min N2 35,0 ml/min
[9] 0,0 °C, 5,00 min N2 35,0 ml/min
Synchronization enabled

CD studies of monomer and polymers P1 and P2

Table S3. CD and UV data of monomer **5** and polymers **P1** and **P2** (all measurements in acetonitrile, see exp. part)

	**CD**		**UV**	
Compound	λ	mdeg	λ	A
Monomer **5**	205<br>232<br>244<br>277<br>335	-51.2<br>+9.4<br>-7.3<br>-2.8<br>-4.8	200<br>231<br>278<br>327<br>336	1.48<br>1.19<br>0.15<br>0.20<br>0.22
Polymer **P2**	213<br>216<br>219<br>231<br>246<br>277<br>327<br>338	-19.1<br>+4.6<br>-7.2<br>+5.3<br>-6.4<br>-1.8<br>-3.0<br>-3.4	 <br>221<br>238<br><br>280<br>329<br>338	 <br>0.78<br>0.79<br><br>0.1<br>0.14<br>0.15
Polymer **P1**	212<br>215<br>219<br>231<br>246<br>328<br>339	-11.7<br>+4.1<br>-7.0<br>+6.8<br>-7.2<br>-3.3<br>-3.7	 <br>221<br>237<br>280<br>329<br>338	 <br>0.86<br>0.89<br>0.11<br>0.15<br>0.17

FL emission spectra

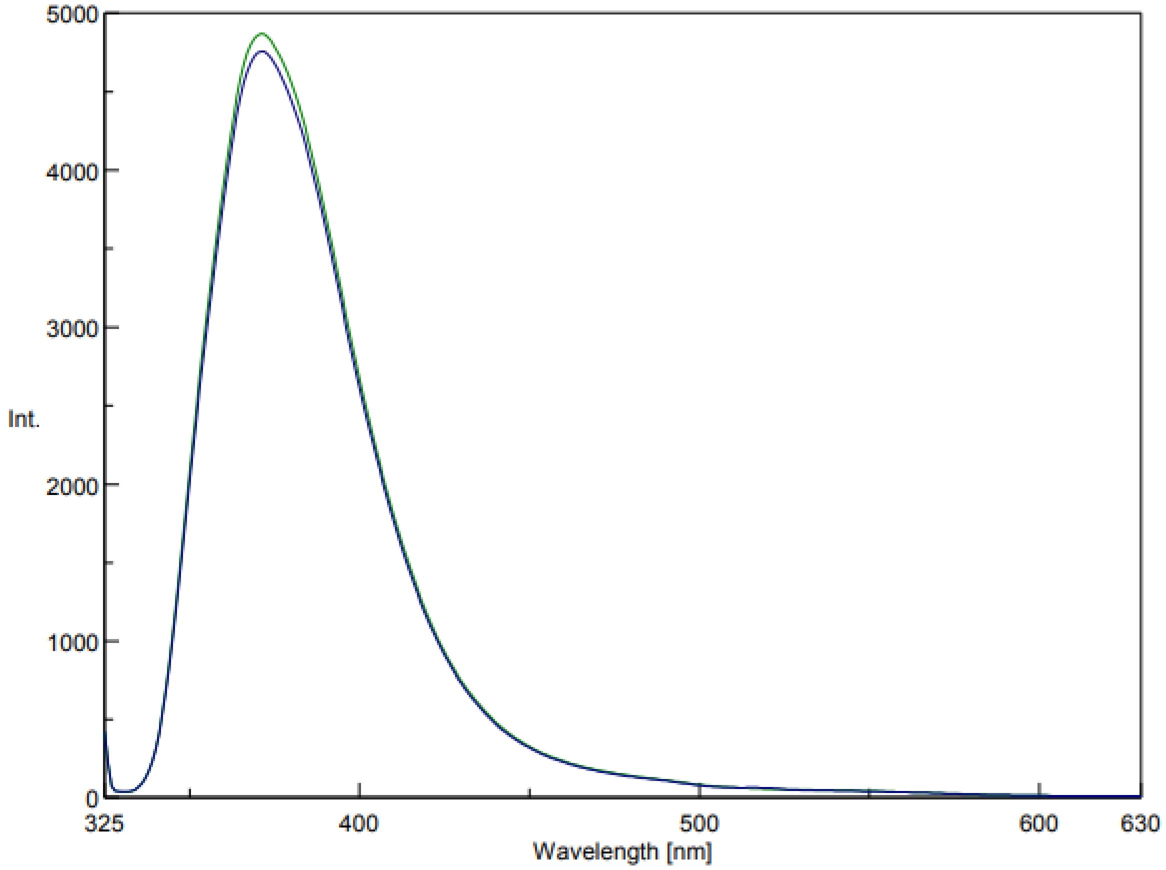

monomer.jws
monomer+(RS).jws

[Comments]
Sample name
Comment
User
Division
Company UAM

[Measurement Information]

Model name	FP-8300
Serial No.	C014561450
Ex filter holder	None
Em filter holder	None
Accessory	SCE-846
Accessory S/N	C014561450
Measurement date	2025-03-14 12:54
Mode	Emission
Ex bandwidth	2.5 nm
Em bandwidth	2.5 nm
Response	1 sec
Sensitivity	High
Measurement range	325 - 630 nm
Data interval	1 nm
Ex wavelength	320.0 nm
Scan speed	500 nm/min
Auto gain	Off
Shutter control	Open only for measurement
Light source	Xe lamp
Filter	Not used
Blank correction	Off

[Detailed Information]

Creation date	2025-03-14 12:54
Data array type	Linear data array
Horizontal axis	Wavelength [nm]
Vertical axis	Int.
Start	325 nm
End	630 nm
Data interval	1 nm
Data points	306

Figure S23. FL emission spectra of monomer **6** (green line), the mixture of monomer **6**/*R,S*-racemic mandelic acids (blue line) and measurement information.

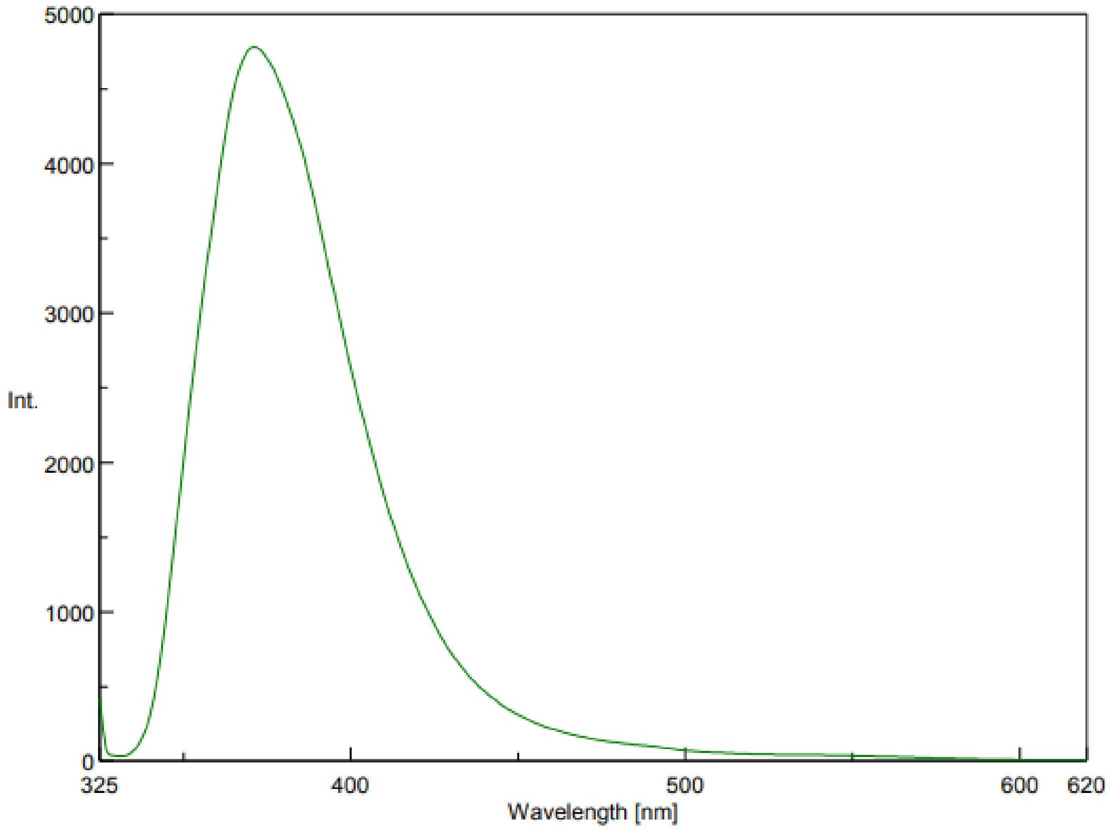

[Comments] P2.jws

Sample name	
Comment	
User	
Division	
Company	UAM

[Measurement Information]

Model name	FP-8300
Serial No.	C014561450
Ex filter holder	None
Em filter holder	None
Accessory	SCE-846
Accessory S/N	C014561450
Measurement date	2025-04-01 10:55
Mode	Emission
Ex bandwidth	2.5 nm
Em bandwidth	2.5 nm
Response	1 sec
Sensitivity	High
Measurement range	325 - 620 nm
Data interval	1 nm
Ex wavelength	320.0 nm
Scan speed	500 nm/min
Auto gain	Off
Shutter control	Open only for measurement
Light source	Xe lamp
Filter	Not used
Blank correction	Off

[Detailed Information]

Creation date	2025-04-01 10:55
Data array type	Linear data array
Horizontal axis	Wavelength [nm]
Vertical axis	Int.
Start	325 nm
End	620 nm
Data interval	1 nm
Data points	296

Figure S24. FL emission spectrum of **P2** (green line) and measurement information.

NMR spectra for general procedure for self-metathesis of quinine

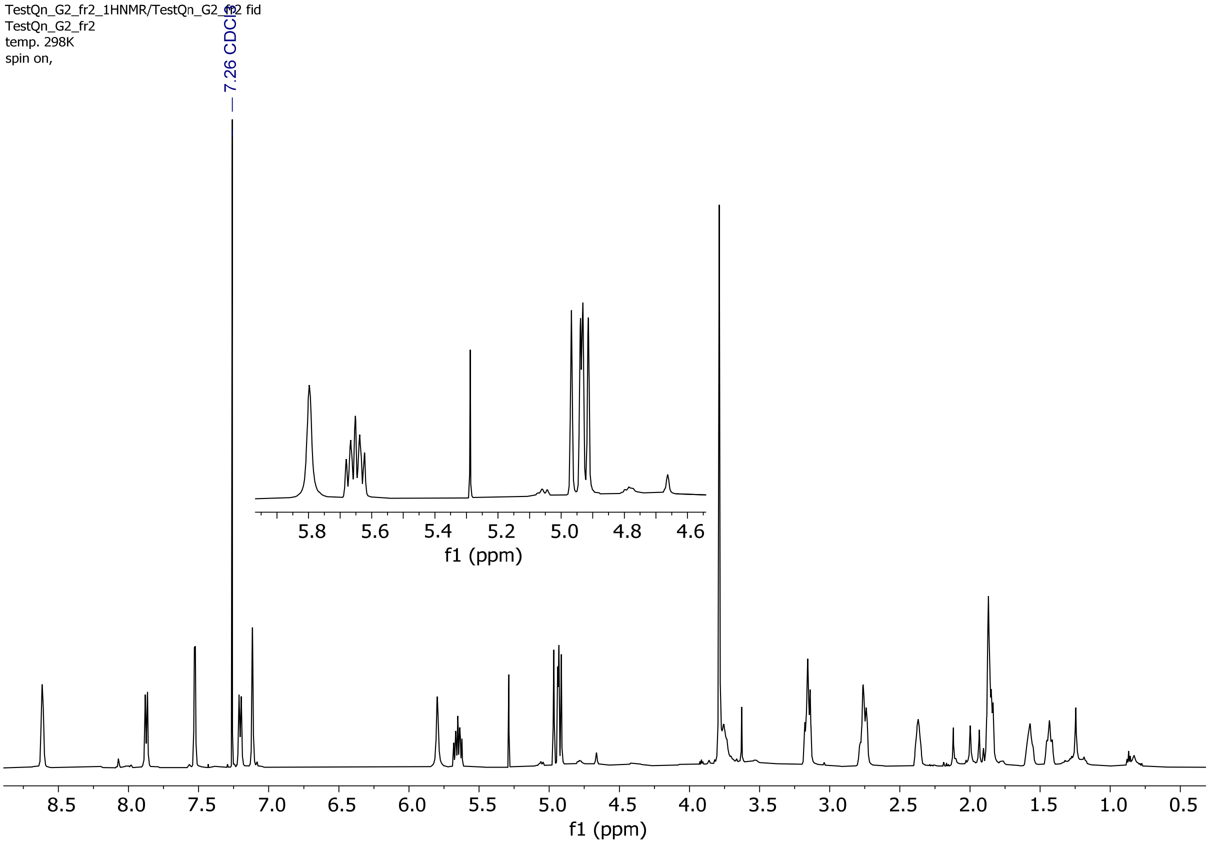

Figure S25. ¹H NMR spectrum of QN in $CDCl_3$ at 25°C.

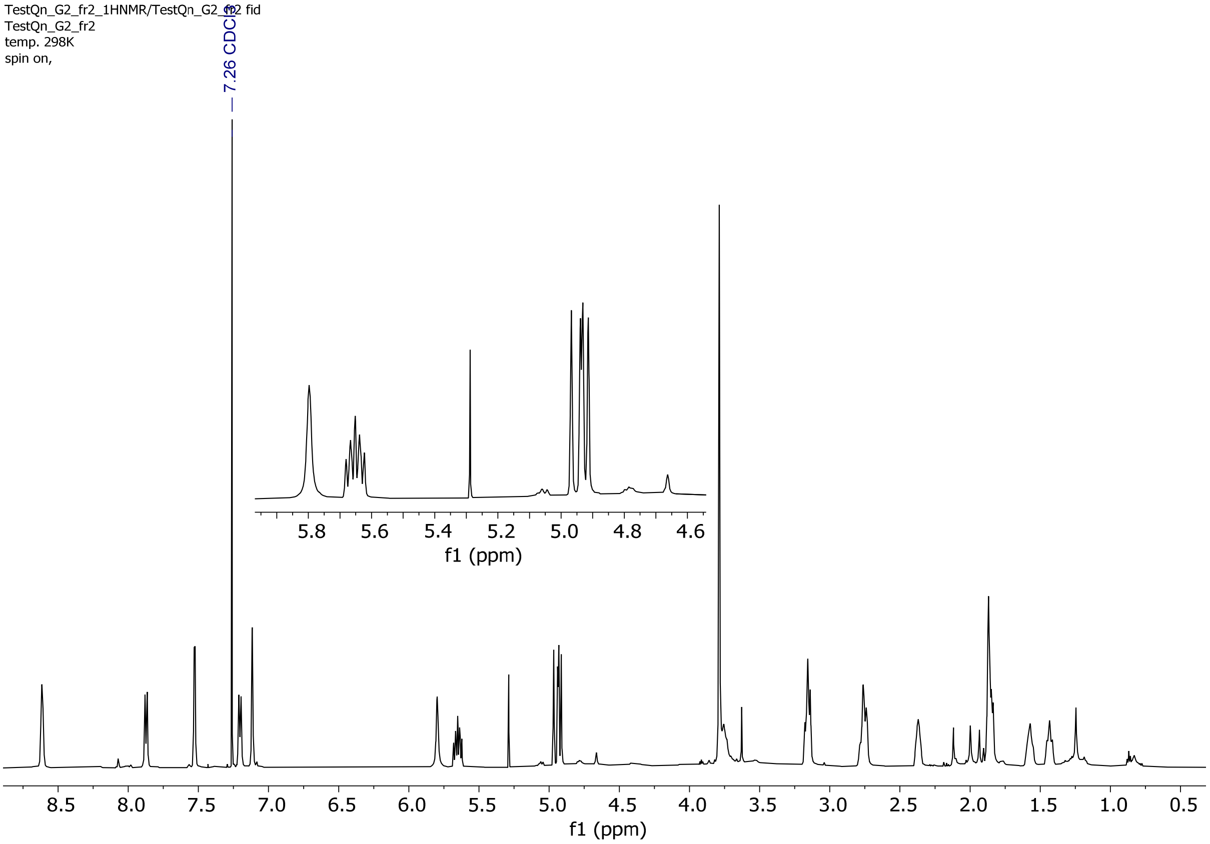

Figure S26. ¹H NMR spectrum of QN + G2 in $CDCl_3$ at 25°C.

References

(1). Choi, T.-L.; Grubbs, R. H. *Angew. Chem. Int. Ed.*, **2003**, (42), 1743-1746.

(2). Bielawski, Ch. W.; Benitez, D.; Morita, T.; Grubbs, R. H. *Macromolecules*, **2001**, (34), 8610-8618.

(3). Schrock, R. R.; Feldman, J.; Cannizzo, L. F.; Grubbs, R. H. *Macromolecules*, **1987**, (20), 20 1169-1172.

(4). Katz, T. J.; Lee, S. J.; Acton, N. *Tetrahedron Lett.,* **1976**, (47), 4247-4250.

(5). Spring, A. M.; Maeda, D.; Ozawa, M.; Odoi, K.; Qiu, F.; Yamamoto, K.; Yokoyama, S. *Polymer (UK),* **2015***, vol. 56,* 189-198.

(6). Majchrzak, M.; Hine, P. J.; Khosravi, E. *Polymer (UK)*, **2012**, (53), 5251-5257.

(7). Kacprzak, K.; Gierczyk, B. *Tetrahedron Asymmetry*, **2010**, (21), 2740–2745.

(8). Kacprzak, K.; Migas, M.; Plutecka, A.; Rychlewska, U.; Gawroński, J. *Heterocycles*, **2005**, vol. 65 (No 7), 1931-1938.

(9). Murov, S. L.; Carmichael, I.; Hug, G. L. Handbook of Photochemistry, 2nd Ed; CRC Press: Boca Raton, Florida, **1993**.
